# Supplementary material for: Elemental pollution and risk assessment of soils and Gundelia tournefortii in a multi-sector industrial zone with a history of agricultural use
Source: PeerJ. 2025 Nov 24;13:e20374. doi: 10.7717/peerj.20374 (PMC12659707; doi:10.7717/peerj.20374)
Supplement: Supplemental Information 1 [file peerj-13-20374-s001.pdf]

Table S1. The main heavy metals and other elements found in industrial emissions

Industries	Polluting Elements	Reference
Agriculture industry	Fertilizers: Aluminum, cadmium, nickel, nitrogen, phosphorus, potassium, zinc<br>Pesticides: Aluminum, arsenic, cadmium, calcium, chlorine, chromium, cobalt, copper, iron, lead, manganese, mercury, nickel, sodium, sulfur, zinc	(Aktaruzzaman et al., 2024; Bradl, 2005; Gimeno-García, Andreu, & Boluda, 1996; Zoffoli et al., 2013)
Automative industry	Arsenic, cadmium, chromium, copper, iron, lead, manganese, mercury, nickel, selenium, vanadium, zinc	(Bradl, 2005; Gautam, Gautam, Banerjee, Chattopadhyaya, & Pandey, 2016)
Chemical industry	Cadmium, chromium, cobalt, lead, manganese, mercury, nickel, copper	(Bradl, 2005; Gautam et al., 2016; Perusic, Vasiljević, & Pelemis, 2019)
Construction materials	Arsenic, cadmium, cobalt, nickel, lead, titanium	(Bradl, 2005; Gautam et al., 2016)
Electric-electronics industry	Antimony, chromium, copper, lead, manganese, mercury, molybdenum, nickel, selenium, thallium, zinc	(Bradl, 2005; Gautam et al., 2016)
Iron and steel processing	Arsenic, cadmium, chromium, iron, lead, manganese, mercury, nickel, zinc, copper	(Bradl, 2005; Dai et al., 2015; Gautam et al., 2016; Perusic et al., 2019)
Machine production	Arsenic, cadmium, chromium, copper, iron, lead, manganese, mercury, nickel, selenium, vanadium, zinc	(Bradl, 2005; Gautam et al., 2016; National Atmospheric Emissions Inventory (NAEI), 1999; Passant et al., 2002)
Paper and paperboard	Mercury, lead, potassium, sulfur	(Bradl, 2005; Dionne & Walker, 2021; Gautam et al., 2016; Jaworska, Matuszczak, & Różański, 2020; Perusic et al., 2019)
Plastic industry	Cadmium, lead, mercury, zinc	(Bradl, 2005; Gautam et al., 2016; Perusic et al., 2019)
