# Supplementary material for: Elemental pollution and risk assessment of soils and Gundelia tournefortii in a multi-sector industrial zone with a history of agricultural use
Source: PeerJ. 2025 Nov 24;13:e20374. doi: 10.7717/peerj.20374 (PMC12659707; doi:10.7717/peerj.20374)
Supplement: Supplemental Information 2 [file peerj-13-20374-s002.pdf]

Table S2. The heavy metal values in soils, crops and other WEPS in previous studies

Site	Media	Cu	Ni	Pb	Zn	Cd	Mn	Cr	Co	Hg	As	Fe	Al	Reference
**Earth Crust Average**	**Soil**	60	84	14	70	0.15	950	102	25	0.085	1.8	56300	82300	(Champion, 2008)
**Iron and steel production area, China**	**Soil**	42.42±23.41	33.76±8.42	82.77±58.40	267.62±123.50	0.3±0.11		91.19±22.98		0.13±0.10	10.64±0.99			(X. Zhang et al., 2022)
**Paper and paperboard production area, Poland**	**Soil**	12.38±7.801	9.05±5.557	0.21±0.972	47.03± 29.586			16.40±9.435		0.04±0.022				(Jaworska, Matuszczak, & Różański, 2020)
	**Plant (Pine Bark)**	1.42-16.61	0-6.20	0-61.81	49.91-105.21			0		0.10-0.32				
**Industrialized city, India**	**Soil**	9.8-19.30		2.9-10.3	83-133	0.6-2.3								(Sharma, Agrawal, & Marshall, 2009)
	**Lady's finger**	9.5-21.8		0.3-1.2	29.6-39.2	0.5-1.2								
	**Cauliflower**	9.8-24.1		0.2-1.8	38.6-63.3	0.6-2.1								
	**Palak**	12.8-25.6		0.7-1.4	30.1-45.50	0.4-1.5								
**Organized Industrial Zone, Turkey**	**Soil**	95.88±157		246 ±1120	632 ±1562	4.41±23.37	1824±2523	118 ±236		0.1±0.37	9.53±9.25			(Yaylali-Abanuz, 2011)
**Industrialized city, Italy**	**Agricultural Soil**	7.3-354	11-303	0.4-46.2	29.3-139.9			9.3-192.8	7.3-34.6					(Facchinelli, Sacchi, & Mallen, 2001)
**Industrialized city, Spain**	**Agricultural Soil**	22.5±8.9	20.9±5.1	22.8±16.1	52.8±14.9	0.34±0.2	295±61	26.5±5.9	7.1±1.7			13608±3107		(Micó, Recatalá, Peris, & Sánchez, 2006)
**Industrialized city, China**	**Soil**	25.76±11.69		26.3±20.82	71.97±26.56	1.47±1.78		75.82±15.32		0.80±0.14	9.33±3.8			(Wang, Duan, & Wang, 2020)
**Industrial Zone, UK**	**Soil**	28.8	27.8	68.9	114	0.38	540	35.7		0.14	12.5			(Alloway, 2012)
**Chemical industry park, China**	**Soil**	466.00±15.40	69.30±4.32	394.00±5.43	1729.00±23.90			248.00±7.03						(T. Zhang et al., 2021)
**Coal chemical industrial area, China**	**Soil**			17.087±1.104		0.553±0.065		93.432±8.663		0.092±0.038	12.840±1.174			(K. Zhang, Qiang, & Liu, 2018)
**Industrial Zone, India**	**Soil**	4-55	6.5-32		14.1-672	0.06-176		103-191	3-11					(Govil, Reddy, & Krishna, 2001)
**Iron and steel production area, Czech Republic**	**Soil**	10.37-101.44		81-5286	40-9288	1.41-63.61								(Friedlova, 2010)
**General**	**Uncontaminated soil**	2–100	0.0–5	2–200	10–300	0.01–0.7	100-4000	5-3000	1–40			7000–55000		(Allaway, 1968)
	**Agricultural crops**	4–15	1.0	0.1–10	15–200	0.2–0.8	15–100	0.2–1.0	0.05–0.5					

Site	Media	Cu	Ni	Pb	Zn	Cd	Mn	Cr	Co	Hg	As	Fe	Al	Reference
**Contaminated field trial**	**Soil**	60.34	61.05	52.69	146.39	25.91								(Singh, Zacharias, Kalpana, & Mishra, 2012)
	**Potato**	26	27	43	96	30								
	**Spinach**	29	11	23	86	20								
	**Fenugreek**	88	5	36	64	4								
	**Cauliflower**	13	40	29	53	3								
	**Okra**	71	7	33	65	7								
**Agricultural area, Spain**	**Soil**	35.4 ± 8.1	19.9 ± 5.2	56.1 ± 84.3	76.8 ± 20.9	0.358 ± 0.221	408 ± 119	32.2 ± 9.3	7.9 ± 2.0			17487 ± 5270		(Peris, Micó, Recatalá, Sánchez, & Sánchez, 2007)
	**Lettuce**	13.2 ± 4.1	3.84 ± 7.24	1.99 ± 1.74	41.7 ± 16.6	1.47 ± 2.16	63 ± 37	3.35 ± 5.54	0.57 ± 0.54			431 ± 692		
	**Soil**	36.0 ± 10.8	19.4 ± 3.1	85.5 ± 251.8	94.5 ± 53.6	0.352 ± 0.154	379 ± 82	32.2 ± 5.2	7.3 ± 1.3			17 373 ± 3 515		
	**Artichoke**	8.7 ± 2.2	1.32 ± 0.92	0.28 ± 0.17	44.3 ± 12.3	0.24 ± 0.12	21 ± 4	0.68 ± 0.72	-			65 ± 43		
**Minin area (Pyrite), China**	**Soil**	271 ±132		190±79	349±201	3.13±1.22								(Zhuang, Zou, Li, & Li, 2009)
	**Rice**	6.34		1.44	34.8	0.82								
	**Vegetables**	10.4		1.53	89	1.99								
**Nonferrous-metal production area, Bulgaria**	**Soil (0.5 km away)**	95.7		200.3	536.1	12.2								(Angelova, Ivanova, & Ivanov, 2004)
	**Sunflower***	5.9 ± 0.03		0.7 ± 0.004	41.9 ± 1.2	0.34 ± 0.06								
	**Sesame***	5.3 ± 0.1		1.9 ± 0.2	26.2 ± 0.1	0.10 ± 0.08								
	**Rapeseed***	4.6 ± 0.05		2.6 ± 0.2	66.9 ± 1.5	0.62 ± 0.02								
	**Peanut***	7.3 ± 0.03		-	13.9 ± 0.2	1.1 ± 0.01								
	**Soil (15 km away)**	16.0		24.6	33.9	2.7								
	**Sunflower***	6.6 ± 0.03		0.2 ± 0.001	21.7 ± 0.1	0.12 ± 0.01								
	**Sesame***	3.8 ± 0.1		0.5 ± 0.04	20.4 ± 0.3	0.04 ± 0.001								
	**Rapeseed***	5.2 ± 0.05		0.7 ± 0.04	33.6 ± 1.0	0.22 ± 0.05								
	**Peanut***	3.7 ± 0.01		-	8.7 ± 0.1	0.1 ± 0.001								
**General, Thailand**	**Thailand Soils**	0.16-350	0.01-0.270	0.1-550	0.1-140	0.01-1.3		0.14-295	0.1-113	0.01-0.27	0.08-124			(Zarcinas, Pongsakul, McLaughlin, & Cozens, 2004)
	**Agricultural soil**	59-348	43.8-68.2	15.5-550	94.8-125	0.01-0.76		28.1-64		0.03-0.14	3-69.8			
	**Cabbage**	0.1-0.4	0.02-0.2	0.01-0.04	1.1-2.1	0.003-0.02		0.01-0.07		0.0002-0.0003	0.06-0.16			
	**Agricultural soil**	1.73-50	0.63-270	1.5-63.8	2.9-71.9	0.004-0.29		1.6-295		0.001-0.063	0.5-108			
	**Corn**	0.1-0.2	0.05-0.09	0.01	1.3-3.2	0.0001-0.002		0.01-0.04		0.0001-0.0004	<0.04			
**General, Malayisia**	**Agricultural soil**	2-49	3-45	11-90	10-136	0.01-0.35		5-64		0.02-0.33	4-39			(Zarcinas, Ishak, McLaughlin, & Cozens, 2004)
	**Cabbage**	0.005-20.3	0.015-0.26	0.0005-0.065	0.5-6.1	0.002-0.046		0.005-0.227		0.00005-0.0019	0.06-0.17			
	**Agricultural soil**	4-58	9-28	15-49	16-63	0.02-0.15		2-48		0.05-0.31	3-61			
	**Corn**	0.32-0.38	0.015-0.11	0.0005-0.01	0.6-2.2	0.002-0.012		0.005-0.021		0.00005-0.00039	0.04-0.08			
**Forest, China**	**Soil**	6.98-103.78		3.21-24.95	52.11-101.04	0.03-5.23	98-2744				2.1-8.61	290-4620		(Liu et al., 2015)
	**Wild edible mushroom**	1.53-35.68		0.48-10.18	8.71-59.53	0.17-2.88	1.54-110.5				0.76-11.86	2.00–826.50		
**Roadsides, Jordan**	**Soil**	33.44±1.5	144.45±2.2	15.33±2.2	42.12±4.1	1.77±1.2	215.74±7.3		25.63±2.1			475.44±9.4		(Semreen & Aboul-Enein, 2011)
	**Wild edible mushroom**	41.81±1.5	2.81±3.1	3.81±1.2	39.56±3.2	2.12±3.3	34.65±2.4		3.5±01			254.68±3.7		

Site	Media	Cu	Ni	Pb	Zn	Cd	Mn	Cr	Co	Hg	As	Fe	Al	Reference
**Industrial Zone, Pakistan**	**Soil**	8.88-357.40	41.4-57.5	2-29				40.24-927.2	7.26-24.73					(Malik, Husain, & Nazir, 2010)
	**Portulaca oleracea Root**	190.7-225.7	2.1-2.2	8-10	26.5-28.6			18.8-26.4	8.3-39.5					
	**Portulaca oleracea Shoot**	35.5-171.8	6.2-9.5	11-19	26.5-28.6			9.8-14.9	17.6-20.2					
**Near waste disposal area, Italy**	**Soil**	104-902	137-891		408-1150	0.95-1.72		459-1770						(Brunetti, Soler-Rovira, Farrag, & Senesi, 2009)
	**Carduus pycnocephalus shoot**	20.0–22.2	1.3–2.5	1.1–1.6	39.9–54.8	0.28–0.53		2.7–4.9						
	**Carduus pycnocephalus root**	12.3–13.6	0.8–0.9	0.9–1.1	23.1–24.4	0.29–0.39		7.3–8.2						
	**Silybum marianum shoot**	10.7–23.4	0.7–5.9	0.4–1.4	22.8–79.5	0.35–0.56		0.6–11.1						
	**Silybum marianum root**	21.3–30.1	0.3–2.6	0.4–1.8	28.4–57.9	0.15–0.35		2.0–11.3						
**Industrial zone, Pakistan**	**Soil**	73.5–576.7	124.8–710	103.7–3001		3.37–90.22		76.93- 677.2						(Ahmad, Gul, Irum, Manzoor, & Arshad, 2023)
	**Roots of wild plants**	11.87–278	8.14–93.8	25.85–670		2.49–45		31.16-245						
	**Shoots of wild plants**	20.5–386	30–79.5	8.77–1244		1.9–75		63.77–580						
**Industrialized city, Türkiye**	**Brook coast soil**	70.69 ± 5.64		73.44 ± 4.85	11.92 ± 1.13	2.35 ± 0.18								(Osma, Ozyigit, Demir, & Yasar, 2014)
	**Brook Coast Wild Portulaca oleracea**	16.78 ± 1.23		5.38 ± 0.92	43.19 ± 3.67	0.36 ± 0.05								
	**Roadside soil**	62.73 ± 5.54		36.68 ± 2.56	140.73 ± 10.56	2.29 ± 0.14								
	**Roadside Wild Portulaca oleracea**	17.03 ± 1.42		5.24 ± 0.92	59.49 ± 3.48	0.29 ± 0.07								
**Metropolitan city, Italy**	**Portulaca oleracea (Countryside)**	20.02±1.52	6.2±2.09	1.22±0.19	68.65±8.9	0.08±0.01	50.43±6.32	0.82±0.3	0.14±0.05			546.1±127.8		(Renna, Cocozza, Gonnella, Abdelrahman, & Santamaria, 2015)
	**Portulaca oleracea (Roadside)**	20.01±0.82	36.26±11.07	1.91±0.65	89.51±13.91	0.07±0.02	37.82±10.93	1.8±1.29	0.16±0.19			423.7±305.9		
**Mountain, Türkiye**	**Eremurus spectabilis**	1.379±0.113	0.279±0.029	0.047±0.001	6.809±0.703	n.d	4.886±0.420	n.d	n.d	n.d	0.008±0.001	49.720±4.35	33.489±2.954	(Ekin, 2022)
