# Supplementary material for: Elemental pollution and risk assessment of soils and Gundelia tournefortii in a multi-sector industrial zone with a history of agricultural use
Source: PeerJ. 2025 Nov 24;13:e20374. doi: 10.7717/peerj.20374 (PMC12659707; doi:10.7717/peerj.20374)
Supplement: Supplemental Information 5 [file peerj-13-20374-s005.pdf]

Table S5. The detection limits of XRF and ICP-MS, mg/kg_{dw}

		Elements														
		**Cd**	**Cr**	**Cu**	**Ni**	**Pb**	**Zn**	**Al**	**Fe**	**K**	**Na**	**Mg**	**Mn**	**P**	**S**	**Ti**
Limit of detection, mg/kg_{dw}*	XRF	-	-	0.4	-	-	0.4	4	0.4	4	810	4	0.4	0.4	0.4	-
	ICP-MS	0.001	0.004	-	0.011	0.003	-	-	-	-	-	-	-	-	-	0.067

* : dw (dry weight)
