# Supplementary material for: Elemental pollution and risk assessment of soils and Gundelia tournefortii in a multi-sector industrial zone with a history of agricultural use
Source: PeerJ. 2025 Nov 24;13:e20374. doi: 10.7717/peerj.20374 (PMC12659707; doi:10.7717/peerj.20374)
Supplement: Supplemental Information 6 [file peerj-13-20374-s006.pdf]

Table S6. pH, EC, and organic matter levels of soil samples

	pH	EC, µs/cm	OM, %dw
**S1**	6.6	430	13.92
**S2**	6.56	181	4.73
**S3**	6.37	216	5.32
**S4**	6.42	288	6.98
**S5**	7.23	558	6.08
**S6**	6.83	245	4.81
**S7**	6.67	314	6.86
**S8**	6.79	208	3.90
**S9**	6.86	157	3.16
**S10**	6.65	425	4.71
**S11**	6.84	228	4.54
**S12**	6.8	301	5.07
**S13**	6.93	202	4.74
**REF-S**	6.82	364	7.91
