# Supplementary material for: Elemental pollution and risk assessment of soils and Gundelia tournefortii in a multi-sector industrial zone with a history of agricultural use
Source: PeerJ. 2025 Nov 24;13:e20374. doi: 10.7717/peerj.20374 (PMC12659707; doi:10.7717/peerj.20374)
Supplement: Supplemental Information 7 [file peerj-13-20374-s007.pdf]

Table S7. The pollution index values (PI)

	Soil Samples												
	S1	S2	S3	S4	S5	S6	S7	S8	S9	S10	S11	S12	S13
**PI**	**6.82**	**2.91**	**4.47**	**3.21**	**2.06**	**4.20**	**2.33**	**2.66**	**2.46**	**3.39**	**3.19**	**2.39**	**2.18**

PI > 1.0 indicates the presence of anthropogenic pollution in the soil
