# Supplementary material for: Elemental pollution and risk assessment of soils and Gundelia tournefortii in a multi-sector industrial zone with a history of agricultural use
Source: PeerJ. 2025 Nov 24;13:e20374. doi: 10.7717/peerj.20374 (PMC12659707; doi:10.7717/peerj.20374)
Supplement: Supplemental Information 12 [file peerj-13-20374-s012.pdf]

Table S12. Bio-concentration and translocation factors of heavy metals and other elements

		Elements														
**Samples**	**Factors**	**Cd**	**Cr**	**Cu**	**Ni**	**Pb**	**Zn**	**Al**	**Fe**	**K**	**Na**	**Mg**	**Mn**	**P**	**S**	**Ti**
	**BCF_r**	0.001	0.014	**3.099**	0.001	0.002	**1.261**	0.101	0.036	**13.214**	**8.339**	**2.114**	0.605	**3.314**	**4.785**	0.00003
**1**	**BCF_s**	0.030	0.036	**1.338**	0.009	0.002	0.364	0.098	0.010	**7.194**	**10.131**	**1.789**	0.309	**1.469**	**7.222**	0.0001
	**TF**	**29.379**	**2.604**	0.432	**6.922**	**1.166**	0.289	0.969	0.279	0.544	**1.215**	0.847	0.511	0.443	**1.509**	**3.327**
	**BCF_r**	0.030	0.017	**4.881**	0.002	0.009	**2.065**	0.095	0.043	**11.794**	**153.093**	**2.209**	**1.132**	**6.379**	**21.493**	0.00003
**2**	**BCF_s**	0.785	0.044	**2.520**	0.014	0.010	**1.298**	0.069	0.031	**8.849**	**121.753**	**1.666**	0.392	**2.634**	**13.154**	0.0001
	**TF**	**26.053**	**2.578**	0.516	**8.278**	**1.213**	0.628	0.731	0.729	0.750	0.795	0.754	0.346	0.413	0.612	**4.331**
	**BCF_r**	0.0004	0.006	**4.182**	0.001	0.005	**2.571**	0.050	0.006	**16.338**	**145.647**	**1.974**	0.494	**7.624**	**10.477**	0.00003
**3**	**BCF_s**	0.041	0.019	**1.972**	0.012	0.007	**1.367**	0.045	0.010	**6.391**	**126.941**	**1.758**	0.245	**2.979**	**7.551**	0.0001
	**TF**	**112.770**	**2.897**	0.472	**9.600**	**1.544**	0.532	0.893	**1.718**	0.391	0.872	0.891	0.495	0.391	0.721	**2.657**
	**BCF_r**	0.0002	0.011	**5.098**	0.002	0.780	**1.537**	0.076	0.025	**15.924**	**152.047**	**2.447**	0.638	**6.459**	**15.600**	0.00003
**4**	**BCF_s**	0.007	0.025	**2.890**	0.020	0.719	0.778	0.084	0.028	**10.195**	**156.023**	**2.440**	0.408	**3.296**	**18.137**	0.0001
	**TF**	**36.600**	**2.315**	0.567	**9.556**	0.923	0.506	**1.105**	**1.119**	0.640	**1.026**	0.997	0.640	0.510	**1.163**	**4.108**
	**BCF_r**	0.029	0.008	**4.789**	0.002	**1.549**	**3.935**	0.081	0.029	**14.710**	**186.824**	**3.104**	0.627	**9.991**	**8.273**	0.00003
**5**	**BCF_s**	0.681	0.015	**2.824**	0.008	**1.608**	**2.570**	0.078	0.020	**11.077**	**170.235**	**2.582**	0.323	**4.420**	**18.084**	0.0001
	**TF**	**23.675**	**1.819**	0.590	**4.417**	**1.038**	0.653	0.970	0.682	0.753	0.911	0.832	0.516	0.442	**2.186**	**4.036**
	**BCF_r**	0.001	0.720	**4.213**	0.007	0.022	**3.841**	0.052	0.021	**20.439**	**46.250**	**1.868**	0.585	**7.877**	**14.997**	0.00004
**6**	**BCF_s**	0.005	0.351	**2.238**	0.006	0.008	**1.818**	0.043	0.008	**9.200**	**39.333**	**1.789**	0.367	**3.817**	**8.749**	0.0001
	**TF**	**3.892**	0.487	0.531	0.823	0.351	0.473	0.829	0.369	0.450	0.850	0.958	0.627	0.485	0.583	**4.122**
	**BCF_r**	0.033	0.644	**3.733**	0.004	**1.278**	**2.620**	0.080	0.030	**16.643**	**88.500**	**2.178**	0.843	**7.443**	**11.684**	0.00003
**7**	**BCF_s**	0.907	0.381	**1.917**	0.010	0.770	0.921	0.100	0.016	**10.099**	**91.938**	**2.254**	0.373	**3.097**	**12.552**	0.0003
	**TF**	**27.341**	0.591	0.513	**2.435**	0.603	0.352	**1.250**	0.532	0.607	**1.039**	**1.035**	0.442	0.416	**1.074**	**9.316**
	**BCF_r**	0.270	0.036	**3.084**	0.010	**1.856**	**3.271**	0.098	0.021	**16.849**	**19.347**	**2.038**	0.522	**6.195**	**22.351**	0.0003
**8**	**BCF_s**	0.501	0.019	**1.264**	0.017	0.766	**2.143**	0.101	0.009	**10.039**	**23.903**	**2.240**	0.252	**2.902**	**31.483**	0.0003
	**TF**	**1.854**	0.534	0.410	**1.797**	0.413	0.655	**1.026**	0.410	0.596	**1.235**	**1.099**	0.482	0.468	**1.409**	**1.218**
	**BCF_r**	0.320	0.041	**4.969**	0.018	**1.224**	**7.022**	0.108	0.097	**21.600**	**146.901**	**2.593**	**1.115**	**7.749**	**15.040**	0.0001
**9**	**BCF_s**	**1.196**	0.012	**1.946**	0.010	0.393	**2.670**	0.113	0.040	**14.182**	**171.696**	**2.426**	0.402	**3.964**	**34.045**	0.0002
	**TF**	**3.742**	0.284	0.392	0.543	0.321	0.380	**1.049**	0.411	0.657	**1.169**	0.935	0.360	0.512	**2.264**	**1.373**

**Samples**	**Factors**	**Elements**														
		**Cd**	**Cr**	**Cu**	**Ni**	**Pb**	**Zn**	**Al**	**Fe**	**K**	**Na**	**Mg**	**Mn**	**P**	**S**	**Ti**
**10**	**BCF_r**	0.005	0.015	**3.524**	0.011	0.856	**5.166**	0.067	0.015	**18.829**	**164.000**	**2.169**	0.882	**8.743**	**24.734**	0.00003
	**BCF_s**	0.006	0.008	**1.943**	0.005	0.247	**1.948**	0.063	0.019	**12.315**	**122.588**	**1.573**	0.376	**3.923**	**13.960**	0.0001
	**TF**	**1.353**	0.551	0.551	0.407	0.289	0.377	0.948	**1.321**	0.654	0.747	0.725	0.426	0.449	0.564	**2.973**
**11**	**BCF_r**	0.004	0.780	**3.015**	0.010	**1.546**	**1.327**	0.093	0.012	**20.668**	**11.553**	**3.156**	0.675	**8.005**	**15.046**	0.0002
	**BCF_s**	0.010	**1.455**	**1.239**	0.012	**1.374**	**1.152**	0.130	0.012	**8.058**	**15.326**	**3.524**	0.324	**2.918**	**16.971**	0.0001
	**TF**	**2.424**	**1.865**	0.411	**1.209**	0.889	0.868	**1.397**	**1.020**	0.390	**1.327**	**1.117**	0.479	0.365	**1.128**	0.882
**12**	**BCF_r**	0.156	0.574	**4.546**	0.016	0.011	**2.391**	0.101	0.018	**17.115**	**17.089**	**2.654**	0.622	**7.667**	**7.079**	0.0001
	**BCF_s**	**1.634**	0.458	**1.900**	0.008	0.008	**1.585**	0.102	0.011	**8.176**	**17.844**	**2.722**	0.318	**3.827**	**9.167**	0.0001
	**TF**	**10.453**	0.797	0.418	0.507	0.721	0.663	**1.008**	0.602	0.478	**1.044**	**1.025**	0.511	0.499	**1.295**	**1.453**
**13**	**BCF_r**	0.188	0.009	**4.313**	0.003	**1.059**	**1.251**	0.131	0.082	**12.746**	**152.900**	**2.530**	0.547	**4.664**	**13.723**	0.0001
	**BCF_s**	0.637	0.021	**1.831**	0.018	0.546	0.382	0.157	0.044	**6.505**	**206.000**	**3.357**	0.263	**3.115**	**15.467**	0.0003
	**TF**	**3.385**	**2.300**	0.425	**5.313**	0.516	0.305	**1.203**	0.538	0.510	**1.347**	**1.327**	0.481	0.668	**1.127**	**2.770**
**Average**	**BCF_r**	0.080	0.221	**4.111**	0.007	0.784	**2.943**	0.087	0.033	**16.682**	**99.422**	**2.387**	0.714	**7.085**	**14.253**	0.0001
	**BCF_s**	0.495	0.219	**1.986**	0.011	0.497	**1.461**	0.091	0.020	**9.406**	**97.978**	**2.317**	0.335	**3.259**	**15.888**	0.0001
	**TF**	**21.763**	**1.509**	0.479	**3.985**	0.768	0.514	**1.029**	0.748	0.571	**1.044**	0.965	0.486	0.466	**1.203**	**3.274**
**St. Dev**	**BCF_r**	0.114	0.321	0.751	0.006	0.697	1.710	0.023	0.027	3.096	68.838	0.408	0.213	1.723	5.982	0.0001
	**BCF_s**	0.538	0.406	0.533	0.005	0.538	0.752	0.032	0.012	2.267	69.304	0.623	0.057	0.757	8.402	0.0001
	**TF**	30.153	0.980	0.069	3.573	0.397	0.173	0.179	0.427	0.122	0.202	0.164	0.085	0.076	0.551	2.184
**St. Dev (%)**	**BCF_r**	142.379	145.327	18.275	86.384	88.903	58.093	25.991	80.293	18.557	69.238	17.111	29.838	24.318	41.973	93.462
	**BCF_s**	108.590	185.788	26.829	40.837	108.328	51.489	35.628	61.503	24.097	70.734	26.884	16.962	23.242	52.886	45.976
	**TF**	138.550	64.937	14.331	89.659	51.706	33.659	17.350	57.068	21.400	19.311	16.975	17.463	16.252	45.787	66.699
