# Supplementary material for: Elemental pollution and risk assessment of soils and Gundelia tournefortii in a multi-sector industrial zone with a history of agricultural use
Source: PeerJ. 2025 Nov 24;13:e20374. doi: 10.7717/peerj.20374 (PMC12659707; doi:10.7717/peerj.20374)
Supplement: Supplemental Information 13 [file peerj-13-20374-s013.pdf]

Table S13. Correlations among the levels of heavy metals and other elements in soil samples

Correlations

		**Cd**	**Cr**	**Cu**	**Ni**	**Pb**	**Zn**	**Al**	**Fe**	**K**	**Na**	**Mg**	**Mn**	**P**	**S**	**Ti**
**Cd**	**r**	1	-0.256	-0.486	**-.628***	0.241	0.276	**-.591***	**-.668***	**-.637***	-0.118	**-.644***	**-.619***	**-.671***	-0.408	**-.655***
	p		0.398	0.092	0.021	0.428	0.362	0.033	0.013	0.019	0.700	0.018	0.024	0.012	0.167	0.015
**Cr**	**r**		1	-0.114	0.239	-0.313	-0.230	0.133	0.164	0.192	**-.621***	0.021	0.188	0.148	-0.062	0.176
	p			0.712	0.432	0.298	0.449	0.665	0.592	0.530	0.023	0.946	0.539	0.629	0.840	0.566
**Cu**	**r**			1	**.824****	**-.643***	**-.694****	**.788****	**.836****	**.722****	0.296	**.924****	**.798****	**.748****	0.056	**.833****
	p				0.001	0.018	0.009	0.001	0.000	0.005	0.326	0.000	0.001	0.003	0.856	0.000
**Ni**	**r**				1	**-.768****	-0.539	**.953****	**.977****	**.941****	-0.108	**.939****	**.943****	**.852****	0.114	**.946****
	p					0.002	0.058	0.000	0.000	0.000	0.725	0.000	0.000	0.000	0.710	0.000
**Pb**	**r**					1	0.344	**-.777****	**-.777****	**-.767****	0.005	**-.709****	**-.722****	**-.772****	-0.199	**-.734****
	p						0.250	0.002	0.002	0.002	0.987	0.007	0.005	0.002	0.515	0.004
**Zn**	**r**						1	-0.466	-0.532	-0.397	0.043	-0.551	**-.624***	-0.319	0.306	**-.558***
	p							0.109	0.061	0.180	0.888	0.051	0.023	0.288	0.310	0.048
**Al**	**r**							1	**.964****	**.980****	-0.048	**.938****	**.965****	**.865****	0.117	**.960****
	p								0.000	0.000	0.876	0.000	0.000	0.000	0.702	0.000
**Fe**	**r**								1	**.957****	0.046	**.948****	**.966****	**.867****	0.178	**.945****
	p									0.000	0.882	0.000	0.000	0.000	0.561	0.000
**K**	**r**									1	-0.100	**.918****	**.951****	**.887****	0.199	**.954****
	p										0.746	0.000	0.000	0.000	0.514	0.000
**Na**	**r**										1	0.110	-0.066	0.060	0.254	-0.072
	p											0.721	0.831	0.847	0.402	0.816
**Mg**	**r**											1	**.921****	**.875****	0.142	**.954****
	p												0.000	0.000	0.643	0.000
**Mn**	**r**												1	**.819****	0.081	**.945****
	p													0.001	0.794	0.000
**P**	**r**													1	0.515	**.899****
	p														0.072	0.000
**S**	**r**														1	0.197
	p															0.519
**Ti**	**r**															1
	p															

**. Correlation is significant at the 0.01 level (2-tailed).
*. Correlation is significant at the 0.05 level (2-tailed).
p shows the statistical significancy of the correlations among the studied parameters
