# Supplementary material for: Elemental pollution and risk assessment of soils and Gundelia tournefortii in a multi-sector industrial zone with a history of agricultural use
Source: PeerJ. 2025 Nov 24;13:e20374. doi: 10.7717/peerj.20374 (PMC12659707; doi:10.7717/peerj.20374)
Supplement: Supplemental Information 14 [file peerj-13-20374-s014.pdf]

Table S14. Correlations among the levels of heavy metals and other elements in root samples

Correlations

		Cd	Cr	Cu	Ni	Pb	Zn	Al	Fe	K	Na	Mg	Mn	P	S	Ti
**Cd**	**r**	1	0.156	-0.550	**.692****	-0.307	-0.494	-0.437	-0.237	**-.636***	**-.665***	**-.658***	**-.553***	**-.646***	-0.491	0.418
	p		0.611	0.052	0.009	0.307	0.086	0.136	0.436	0.019	0.013	0.015	0.050	0.017	0.089	0.155
**Cr**	**r**		1	-0.219	**.685****	-0.046	-0.507	-0.547	-0.088	-0.071	**-.557***	**-.569***	-0.255	-0.188	-0.529	0.312
	p			0.473	0.010	0.881	0.077	0.053	0.775	0.817	0.048	0.042	0.400	0.538	0.063	0.299
**Cu**	**r**			1	-0.418	0.361	**.603***	-0.142	-0.225	**.617***	0.352	0.402	**.808****	**.682***	0.297	**-.661***
	p				0.155	0.225	0.029	0.644	0.460	0.025	0.238	0.173	0.001	0.010	0.325	0.014
**Ni**	**r**				1	-0.430	**-.743****	**-.580***	-0.306	-0.408	**-.644***	**-.660***	-0.460	-0.374	**-.662***	0.453
	p					0.142	0.004	0.038	0.310	0.167	0.018	0.014	0.113	0.208	0.014	0.120
**Pb**	**r**					1	0.526	-0.170	-0.450	0.546	0.226	0.375	0.113	0.286	0.258	-0.148
	p						0.065	0.578	0.123	0.053	0.457	0.206	0.713	0.344	0.395	0.630
**Zn**	**r**						1	0.009	0.032	0.545	0.320	0.342	0.409	0.443	**.608***	**-.773****
	p							0.976	0.917	0.054	0.287	0.252	0.165	0.130	0.027	0.002
**Al**	**r**							1	0.532	-0.116	**.612***	**.599***	0.008	-0.027	0.208	0.188
	p								0.061	0.705	0.026	0.031	0.980	0.930	0.496	0.538
**Fe**	**r**								1	-0.466	-0.084	-0.117	0.071	-0.310	0.026	-0.092
	p									0.109	0.786	0.703	0.819	0.303	0.932	0.766
**K**	**r**									1	**.555***	0.498	0.340	**.806****	0.407	-0.430
	p										0.049	0.084	0.255	0.001	0.167	0.142
**Na**	**r**										1	**.945****	0.370	**.694****	0.429	-0.275
	p											0.000	0.213	0.009	0.144	0.363
**Mg**	**r**											1	0.376	**.647***	0.378	-0.224
	p												0.206	0.017	0.203	0.462
**Mn**	**r**												1	**.608***	0.435	**-.659***
	p													0.028	0.137	0.014
**P**	**r**													1	0.286	**-.594***
	p														0.343	0.032
**S**	**r**														1	-0.517
	p															0.071
**Ti**	**r**															1
	p															

**. Correlation is significant at the 0.01 level (2-tailed).
*. Correlation is significant at the 0.05 level (2-tailed).
p shows the statistical significancy of the correlations among the studied parameters
