# Supplementary material for: Elemental pollution and risk assessment of soils and Gundelia tournefortii in a multi-sector industrial zone with a history of agricultural use
Source: PeerJ. 2025 Nov 24;13:e20374. doi: 10.7717/peerj.20374 (PMC12659707; doi:10.7717/peerj.20374)
Supplement: Supplemental Information 15 [file peerj-13-20374-s015.pdf]

Table S15. Correlations among the levels of heavy metals and other elements in stem samples

Correlations

		Cd	Cr	Cu	Ni	Pb	Zn	Al	Fe	K	Na	Mg	Mn	P	S	Ti
**Cd**	**r**	1	0.118	0.045	-0.139	**.608***	0.490	**-.566***	-0.377	-0.465	-0.492	-0.423	-0.059	-0.320	-0.502	**-.629***
	p		0.702	0.885	0.651	0.027	0.089	0.044	0.204	0.110	0.088	0.150	0.849	0.287	0.081	0.021
**Cr**	**r**		1	**-.678***	0.442	**.660***	0.173	0.002	-0.065	**-.735****	-0.058	-0.061	**-.702****	**-.636***	-0.227	-0.372
	p			0.011	0.131	0.014	0.573	0.996	0.833	0.004	0.852	0.844	0.007	0.019	0.456	0.211
**Cu**	**r**			1	**-.643***	-0.369	0.266	-0.509	0.004	**.784****	-0.370	-0.218	**.966****	**.738****	-0.296	-0.008
	p				0.018	0.215	0.379	0.076	0.989	0.002	0.214	0.474	0.000	0.004	0.326	0.980
**Ni**	**r**				1	0.075	-0.319	0.197	0.174	-0.411	-0.015	-0.039	**-.603***	**-.649***	0.049	0.374
	p					0.808	0.289	0.518	0.570	0.163	0.961	0.900	0.029	0.016	0.874	0.209
**Pb**	**r**					1	**.586***	-0.426	-0.542	**-.591***	-0.323	-0.332	-0.474	**-.649***	-0.279	**-.675***
	p						0.035	0.146	0.056	0.034	0.281	0.268	0.101	0.016	0.356	0.011
**Zn**	**r**						1	**-.801****	**-.602***	0.011	**-.600***	-0.550	0.238	-0.072	-0.337	**-.661***
	p							0.001	0.030	0.971	0.030	0.051	0.433	0.816	0.260	0.014
**Al**	**r**							1	0.291	-0.212	**.906****	**.808****	-0.440	-0.029	0.477	0.518
	p								0.335	0.487	0.000	0.001	0.132	0.925	0.099	0.070
**Fe**	**r**								1	0.160	-0.070	-0.108	-0.025	0.178	0.155	0.285
	p									0.603	0.819	0.725	0.934	0.561	0.613	0.345
**K**	**r**									1	-0.157	-0.143	**.819****	**.742****	0.198	0.323
	p										0.607	0.642	0.001	0.004	0.517	0.282
**Na**	**r**										1	**.929****	-0.291	0.064	0.400	0.436
	p											0.000	0.335	0.836	0.176	0.137
**Mg**	**r**											1	-0.162	0.180	0.094	0.448
	p												0.598	0.556	0.759	0.125
**Mn**	**r**												1	**.800****	-0.176	0.041
	p													0.001	0.565	0.894
**P**	**r**													1	-0.031	0.153
	p														0.921	0.617
**S**	**r**														1	0.160
	p															0.601
**Ti**	**r**															1
	p															

**. Correlation is significant at the 0.01 level (2-tailed).
*. Correlation is significant at the 0.05 level (2-tailed).
p shows the statistical significancy of the correlations among the studied parameters
