# Supplementary material for: Elemental pollution and risk assessment of soils and Gundelia tournefortii in a multi-sector industrial zone with a history of agricultural use
Source: PeerJ. 2025 Nov 24;13:e20374. doi: 10.7717/peerj.20374 (PMC12659707; doi:10.7717/peerj.20374)
Supplement: Supplemental Information 16 [file peerj-13-20374-s016.pdf]

Table S16. Correlations among the levels of heavy metals and other elements in soil and root samples

Correlations

		Ro-Cd	Ro-Cr	Ro-Cu	Ro-Ni	Ro-Pb	Ro-Zn	Ro-Al	Ro-Fe	Ro-K	Ro-Na	Ro-Mg	Ro-Mn	Ro-P	Ro-S	Ro-Ti
**S-Cd**	**r**	0.026	-0.171	0.275	-0.140	0.529	0.509	-0.479	**-.569***	0.539	0.006	0.065	0.027	0.283	0.460	-0.379
	p	0.933	0.577	0.363	0.649	0.063	0.075	0.097	0.042	0.057	0.984	0.832	0.931	0.349	0.114	0.202
**S-Cr**	**r**	-0.285	-0.184	0.013	-0.323	-0.242	-0.040	0.326	0.423	-0.035	0.040	0.012	0.319	0.031	0.217	0.108
	p	0.345	0.548	0.967	0.282	0.426	0.897	0.277	0.150	0.910	0.898	0.969	0.289	0.919	0.477	0.725
**S-Cu**	**r**	0.380	**.761****	-0.469	**.787****	-0.406	**-.879****	-0.260	0.000	-0.467	-0.403	-0.426	-0.245	-0.301	**-.612***	0.507
	p	0.201	0.003	0.106	0.001	0.168	0.000	0.391	0.999	0.108	0.173	0.146	0.420	0.317	0.026	0.077
**S-Ni**	**r**	-0.003	**.628***	-0.343	0.405	-0.426	**-.730****	0.062	0.289	-0.251	-0.108	-0.211	-0.027	-0.077	-0.468	0.394
	p	0.992	0.021	0.252	0.170	0.147	0.005	0.841	0.339	0.407	0.726	0.490	0.930	0.802	0.107	0.183
**S-Pb**	**r**	0.065	-0.465	0.533	-0.328	0.351	**.652***	-0.120	-0.159	0.096	-0.002	0.070	0.227	0.021	0.144	-0.440
	p	0.833	0.109	0.061	0.273	0.240	0.016	0.696	0.604	0.754	0.995	0.819	0.455	0.945	0.640	0.133
**S-Zn**	**r**	-0.402	**-.606***	0.105	**-.734****	0.311	**.652***	0.521	0.280	0.204	0.480	0.497	0.016	0.086	**.567***	-0.390
	p	0.173	0.028	0.733	0.004	0.301	0.016	0.068	0.355	0.504	0.097	0.084	0.959	0.779	0.043	0.188
**S-Al**	**r**	0.000	**.565***	-0.340	0.437	-0.480	**-.755****	0.126	0.213	-0.201	-0.021	-0.129	-0.102	-0.020	-0.514	0.436
	p	0.999	0.044	0.256	0.135	0.097	0.003	0.683	0.486	0.510	0.945	0.675	0.739	0.949	0.072	0.137
**S-Fe**	**r**	0.019	**.619***	-0.418	0.453	-0.438	**-.775****	0.109	0.221	-0.243	-0.056	-0.171	-0.149	-0.083	-0.511	0.483
	p	0.951	0.024	0.155	0.120	0.134	0.002	0.724	0.469	0.424	0.856	0.576	0.626	0.788	0.074	0.095
**S-K**	**r**	-0.102	0.484	-0.309	0.333	-0.515	**-.677***	0.207	0.284	-0.166	0.074	-0.070	-0.019	0.041	-0.387	0.345
	p	0.741	0.094	0.304	0.266	0.071	0.011	0.497	0.347	0.587	0.809	0.820	0.950	0.895	0.191	0.248
**S-Na**	**r**	0.437	0.173	**-.580***	0.421	0.080	-0.327	-0.002	-0.231	-0.371	-0.166	-0.090	**-.740****	-0.454	-0.343	0.493
	p	0.135	0.572	0.038	0.152	0.795	0.276	0.994	0.447	0.212	0.589	0.770	0.004	0.119	0.251	0.087
**S-Mg**	**r**	0.156	**.647***	-0.403	**.586***	-0.514	**-.837****	0.005	0.193	-0.395	-0.189	-0.277	-0.114	-0.197	-0.517	0.454
	p	0.611	0.017	0.172	0.035	0.072	0.000	0.987	0.527	0.181	0.536	0.359	0.710	0.519	0.071	0.119
**S-Mn**	**r**	0.053	**.607***	-0.304	0.519	-0.524	**-.773****	0.029	0.133	-0.169	-0.081	-0.218	-0.100	-0.012	**-.553***	0.449
	p	0.863	0.028	0.313	0.069	0.066	0.002	0.926	0.665	0.582	0.794	0.475	0.744	0.969	0.050	0.124
**S-P**	**r**	-0.042	0.448	-0.442	0.394	**-.672***	**-.671***	0.243	0.481	-0.390	-0.032	-0.130	-0.108	-0.100	-0.364	0.273
	p	0.892	0.124	0.130	0.183	0.012	0.012	0.424	0.096	0.188	0.916	0.671	0.725	0.745	0.221	0.367
**S-S**	**r**	-0.417	-0.080	-0.127	-0.141	-0.310	0.017	0.511	0.483	-0.087	0.375	0.348	-0.042	0.115	0.095	-0.161
	p	0.157	0.795	0.679	0.645	0.302	0.957	0.074	0.094	0.777	0.207	0.244	0.892	0.708	0.757	0.600
**S-Ti**	**r**	0.000	0.524	-0.257	0.463	-0.545	**-.764****	0.095	0.223	-0.275	-0.017	-0.104	0.053	0.027	-0.485	0.347
	p	1.000	0.066	0.396	0.111	0.054	0.002	0.756	0.464	0.363	0.957	0.736	0.863	0.931	0.093	0.246

**. Correlation is significant at the 0.01 level (2-tailed).

*. Correlation is significant at the 0.05 level (2-tailed).

p shows the statistical significancy of the correlations among the studied parameters
