# Supplementary material for: Elemental pollution and risk assessment of soils and Gundelia tournefortii in a multi-sector industrial zone with a history of agricultural use
Source: PeerJ. 2025 Nov 24;13:e20374. doi: 10.7717/peerj.20374 (PMC12659707; doi:10.7717/peerj.20374)
Supplement: Supplemental Information 17 [file peerj-13-20374-s017.pdf]

Table S17. Correlations among the levels of heavy metals and other elements in soil and stem samples

Correlations

		**St-Cd**	**St-Cr**	**St-Cu**	**St-Ni**	**St-Pb**	**St-Zn**	**St-Al**	**St-Fe**	**St-K**	**St-Na**	**St-Mg**	**St-Mn**	**St-P**	**St-S**	**St-Ti**
**S-Cd**	**r**	0.143	0.327	0.272	-0.189	0.306	**.593***	-0.499	-0.360	0.004	-0.313	-0.211	0.337	0.046	-0.269	-0.520
	p	0.641	0.275	0.369	0.536	0.309	0.033	0.083	0.227	0.989	0.298	0.489	0.260	0.882	0.374	0.069
**S-Cr**	**r**	-0.288	0.091	-0.141	0.384	-0.222	-0.310	-0.012	**.655***	0.191	-0.276	-0.424	-0.124	-0.003	0.318	0.090
	p	0.340	0.767	0.645	0.196	0.465	0.303	0.969	0.015	0.532	0.361	0.149	0.686	0.991	0.289	0.769
**S-Cu**	**r**	-0.531	-0.519	0.230	-0.097	**-.554***	**-.652***	**.557***	0.227	0.411	**.573***	**.627***	0.177	0.325	0.161	**.697****
	p	0.062	0.069	0.451	0.751	0.050	0.016	0.048	0.455	0.163	0.041	0.022	0.563	0.278	0.600	0.008
**S-Ni**	**r**	**-.608***	-0.333	0.083	0.156	**-.567***	**-.709****	0.476	0.513	0.379	0.334	0.395	0.029	0.305	0.099	**.785****
	p	0.027	0.266	0.788	0.612	0.043	0.007	0.100	0.073	0.201	0.265	0.181	0.924	0.310	0.749	0.001
**S-Pb**	**r**	**.920****	0.085	0.073	-0.187	0.543	**.656***	**-.603***	-0.474	-0.388	-0.477	-0.465	0.015	-0.299	-0.413	**-.622***
	p	0.000	0.783	0.812	0.540	0.055	0.015	0.029	0.102	0.190	0.100	0.109	0.961	0.321	0.161	0.023
**S-Zn**	**r**	0.246	**.587***	-0.513	0.228	0.459	0.380	-0.099	-0.147	**-.588***	-0.191	-0.253	-0.465	-0.523	-0.023	-0.339
	p	0.418	0.035	0.073	0.454	0.115	0.200	0.748	0.631	0.035	0.533	0.405	0.109	0.067	0.939	0.257
**S-Al**	**r**	**-.604***	-0.289	0.051	0.125	-0.548	**-.661***	0.520	0.468	0.326	0.375	0.490	0.012	0.357	0.040	**.706****
	p	0.029	0.338	0.868	0.683	0.053	0.014	0.068	0.107	0.278	0.206	0.089	0.968	0.231	0.896	0.007
**S-Fe**	**r**	**-.632***	-0.340	0.017	0.143	-0.546	**-.701****	**.554***	0.388	0.357	0.450	0.503	-0.014	0.314	0.148	**.795****
	p	0.020	0.256	0.956	0.642	0.054	0.008	0.049	0.190	0.231	0.123	0.079	0.964	0.297	0.629	0.001
**S-K**	**r**	**-.615***	-0.311	0.044	0.147	**-.562***	**-.643***	0.486	0.492	0.369	0.311	0.395	0.019	0.355	0.084	**.735****
	p	0.025	0.301	0.887	0.632	0.046	0.018	0.092	0.088	0.215	0.302	0.182	0.952	0.233	0.786	0.004
**S-Na**	**r**	-0.097	-0.026	-0.335	-0.107	0.136	-0.045	0.519	**-.603***	-0.256	**.753****	**.669***	-0.295	-0.221	0.294	0.111
	p	0.752	0.934	0.263	0.727	0.659	0.884	0.069	0.029	0.398	0.003	0.012	0.329	0.467	0.329	0.718
**S-Mg**	**r**	**-.561***	-0.451	0.121	0.074	**-.592***	**-.718****	**.566***	0.403	0.369	0.459	0.533	0.070	0.298	0.108	**.789****
	p	0.046	0.122	0.694	0.809	0.033	0.006	0.044	0.172	0.215	0.115	0.061	0.819	0.323	0.725	0.001
**S-Mn**	**r**	**-.556***	-0.417	0.148	0.080	**-.610***	**-.672***	0.469	0.409	0.418	0.368	0.478	0.126	0.463	0.038	**.729****
	p	0.049	0.156	0.630	0.794	0.027	0.012	0.106	0.165	0.155	0.216	0.098	0.683	0.111	0.901	0.005
**S-P**	**r**	**-.665***	-0.426	-0.008	-0.022	**-.667***	**-.748****	**.681***	**.563***	0.354	0.497	0.462	-0.001	0.366	0.414	**.679***
	p	0.013	0.147	0.980	0.942	0.013	0.003	0.010	0.045	0.235	0.084	0.112	0.998	0.219	0.160	0.011
**S-S**	**r**	-0.154	-0.160	-0.334	-0.147	-0.125	-0.340	0.474	0.109	-0.086	0.374	0.173	-0.258	0.019	**.639***	0.101
	p	0.615	0.602	0.265	0.633	0.684	0.255	0.101	0.722	0.780	0.208	0.572	0.394	0.951	0.019	0.741
**S-Ti**	**r**	-0.542	-0.407	0.148	-0.013	-0.551	**-.693****	0.492	0.522	0.407	0.340	0.410	0.084	0.394	0.110	**.649***
	p	0.056	0.167	0.630	0.968	0.051	0.009	0.087	0.068	0.167	0.256	0.164	0.786	0.183	0.720	0.016

**. Correlation is significant at the 0.01 level (2-tailed).
*. Correlation is significant at the 0.05 level (2-tailed).
p shows the statistical significancy of the correlations among the studied parameters
