# Supplementary material for: Elemental pollution and risk assessment of soils and Gundelia tournefortii in a multi-sector industrial zone with a history of agricultural use
Source: PeerJ. 2025 Nov 24;13:e20374. doi: 10.7717/peerj.20374 (PMC12659707; doi:10.7717/peerj.20374)
Supplement: Supplemental Information 18 [file peerj-13-20374-s018.pdf]

Table S18. Correlations among the levels of heavy metals and other elements in root and stem samples

Correlations

		St-Cd	St-Cr	St-Cu	St-Ni	St-Pb	St-Zn	St-Al	St-Fe	St-K	St-Na	St-Mg	St-Mn	St-P	St-S	St-Ti
**Ro-Cd**	r	-0.081	-0.200	0.201	-0.246	-0.318	-0.140	0.308	-0.081	0.017	0.484	0.534	0.192	0.191	-0.177	0.160
	p	0.792	0.513	0.510	0.418	0.290	0.647	0.306	0.792	0.955	0.093	0.060	0.530	0.532	0.562	0.603
**Ro-Cr**	r	-0.399	**-.579***	0.370	-0.025	-0.466	-0.310	0.176	-0.058	0.507	0.288	0.388	0.368	0.338	0.132	**.598***
	p	0.176	0.038	0.213	0.935	0.109	0.303	0.564	0.852	0.077	0.340	0.190	0.216	0.259	0.668	0.031
**Ro-Cu**	r	**.705****	-0.017	0.368	-0.191	0.444	0.487	**-.798****	-0.104	0.024	**-.821****	**-.703****	0.277	0.012	-0.421	**-.573***
	p	0.007	0.956	0.217	0.533	0.129	0.091	0.001	0.734	0.937	0.001	0.007	0.359	0.968	0.152	0.041
**Ro-Ni**	r	-0.318	**-.635***	0.439	-0.344	**-.579***	-0.380	0.404	-0.060	0.426	**.576***	**.679***	0.456	0.530	0.063	0.407
	p	0.290	0.020	0.133	0.250	0.038	0.200	0.171	0.846	0.146	0.039	0.011	0.117	0.062	0.838	0.168
**Ro-Pb**	r	0.355	0.519	-0.138	0.135	**.819****	**.636***	-0.548	**-.628***	-0.283	-0.358	-0.329	-0.203	-0.511	-0.254	-0.423
	p	0.234	0.069	0.654	0.661	0.001	0.019	0.052	0.022	0.349	0.229	0.272	0.507	0.074	0.403	0.150
**Ro-Zn**	r	0.526	0.268	0.051	-0.110	0.518	**.789****	**-.744****	-0.303	-0.145	**-.707****	**-.757****	0.071	-0.204	-0.142	**-.625***
	p	0.065	0.376	0.869	0.720	0.070	0.001	0.004	0.314	0.637	0.007	0.003	0.818	0.503	0.644	0.022
**Ro-Al**	r	-0.039	0.510	**-.824****	0.497	0.209	-0.320	0.423	0.313	**-.583***	0.141	0.003	**-.811****	-0.493	0.249	0.016
	p	0.900	0.075	0.001	0.084	0.494	0.286	0.150	0.298	0.037	0.645	0.992	0.001	0.087	0.413	0.960
**Ro-Fe**	r	-0.212	-0.093	-0.325	0.263	-0.358	-0.365	0.312	**.644***	-0.075	0.001	-0.184	-0.320	-0.150	0.437	0.297
	p	0.488	0.762	0.278	0.386	0.230	0.220	0.299	0.018	0.807	0.998	0.547	0.287	0.624	0.136	0.324
**Ro-K**	r	0.186	0.238	0.173	-0.022	0.381	0.480	**-.588***	-0.358	0.098	-0.490	-0.353	0.223	0.142	-0.286	-0.331
	p	0.544	0.433	0.572	0.943	0.199	0.097	0.035	0.229	0.749	0.089	0.236	0.464	0.644	0.344	0.269
**Ro-Na**	r	0.145	0.516	-0.352	0.060	0.497	0.118	-0.086	-0.057	-0.312	-0.176	-0.172	-0.363	-0.174	-0.135	-0.276
	p	0.637	0.071	0.239	0.847	0.084	0.700	0.780	0.855	0.299	0.565	0.574	0.223	0.570	0.661	0.361
**Ro-Mg**	r	0.245	**.611***	-0.428	0.043	**.681***	0.180	-0.106	-0.105	-0.433	-0.185	-0.207	-0.478	-0.337	-0.076	-0.464
	p	0.420	0.027	0.144	0.889	0.010	0.557	0.730	0.733	0.139	0.546	0.497	0.099	0.260	0.806	0.110
**Ro-Mn**	r	0.387	-0.070	0.377	-0.117	0.211	0.198	**-.628***	0.322	0.219	**-.800****	**-.771****	0.254	0.015	-0.262	-0.285
	p	0.192	0.821	0.205	0.704	0.490	0.518	0.021	0.284	0.472	0.001	0.002	0.403	0.962	0.387	0.345
**Ro-P**	r	0.160	0.075	0.336	-0.388	0.314	0.399	-0.531	-0.092	0.266	-0.504	-0.406	0.304	0.347	-0.234	-0.408
	p	0.602	0.809	0.262	0.190	0.297	0.176	0.062	0.764	0.380	0.079	0.169	0.313	0.245	0.442	0.166
**Ro-S**	r	0.059	0.335	-0.098	0.257	0.233	0.269	-0.357	0.028	-0.041	-0.466	**-.571***	-0.010	-0.296	0.089	-0.209
	p	0.847	0.264	0.750	0.398	0.443	0.374	0.231	0.929	0.893	0.108	0.042	0.975	0.325	0.772	0.493
**Ro-Ti**	r	-0.388	0.135	-0.480	0.468	-0.131	-0.539	**.666***	-0.008	-0.225	**.673***	**.665***	-0.466	-0.229	0.198	0.413
	p	0.190	0.661	0.097	0.106	0.669	0.057	0.013	0.979	0.459	0.012	0.013	0.109	0.451	0.517	0.161

**. Correlation is significant at the 0.01 level (2-tailed).

*. Correlation is significant at the 0.05 level (2-tailed).

p shows the statistical significancy of the correlations among the studied parameters
