# Supplementary material for: Elemental pollution and risk assessment of soils and Gundelia tournefortii in a multi-sector industrial zone with a history of agricultural use
Source: PeerJ. 2025 Nov 24;13:e20374. doi: 10.7717/peerj.20374 (PMC12659707; doi:10.7717/peerj.20374)
Supplement: Supplemental Information 19 [file peerj-13-20374-s019.pdf]

Table S19. Total Variance Explained by PCA for soil samples

Component	Initial Eigenvalues			Extraction Sums of Squared Loadings			Rotation Sums of Squared Loadings		
	Total	% of Variance	Cumulative %	Total	% of Variance	Cumulative %	Total	% of Variance	Cumulative %
**1**	7.732	70.291	70.291	7.732	70.291	70.291	7.719	70.170	70.170
**2**	1.540	13.999	84.289	1.540	13.999	84.289	1.553	14.120	84.289
**3**	0.771	7.007	91.297						
**4**	0.506	4.603	95.900						
**5**	0.192	1.750	97.649						
**6**	0.129	1.175	98.824						
**7**	0.066	0.603	99.427						
**8**	0.042	0.384	99.810						
**9**	0.012	0.108	99.919						
**10**	0.007	0.062	99.981						
**11**	0.002	0.019	100.000						
