# Supplementary material for: Elemental pollution and risk assessment of soils and Gundelia tournefortii in a multi-sector industrial zone with a history of agricultural use
Source: PeerJ. 2025 Nov 24;13:e20374. doi: 10.7717/peerj.20374 (PMC12659707; doi:10.7717/peerj.20374)
Supplement: Supplemental Information 21 [file peerj-13-20374-s021.pdf]

Table S21. Total Variance Explained by PCA for root samples

Component	Initial Eigenvalues			Extraction Sums of Squared Loadings			Rotation Sums of Squared Loadings		
	Total	% of Variance	Cumulative %	Total	% of Variance	Cumulative %	Total	% of Variance	Cumulative %
**1**	5.289	52.892	52.892	5.289	52.892	52.892	3.236	32.359	32.359
**2**	1.563	15.626	68.518	1.563	15.626	68.518	2.790	27.898	60.256
**3**	1.065	10.653	79.171	1.065	10.653	79.171	1.891	18.915	79.171
**4**	0.663	6.628	85.799						
**5**	0.569	5.688	91.487						
**6**	0.440	4.405	95.892						
**7**	0.225	2.245	98.138						
**8**	0.106	1.059	99.197						
**9**	0.052	0.519	99.716						
**10**	0.028	0.284	100.000						
