# Supplementary material for: Elemental pollution and risk assessment of soils and Gundelia tournefortii in a multi-sector industrial zone with a history of agricultural use
Source: PeerJ. 2025 Nov 24;13:e20374. doi: 10.7717/peerj.20374 (PMC12659707; doi:10.7717/peerj.20374)
Supplement: Supplemental Information 23 [file peerj-13-20374-s023.pdf]

Table S23. Total Variance Explained by PCA for stem samples

Component	Initial Eigenvalues			Extraction Sums of Squared Loadings			Rotation Sums of Squared Loadings		
	Total	% of Variance	Cumulative %	Total	% of Variance	Cumulative %	Total	% of Variance	Cumulative %
**1**	4.608	41.889	41.889	4.608	41.889	41.889	4.607	41.882	41.882
**2**	3.858	35.071	76.960	3.858	35.071	76.960	3.859	35.078	76.960
**3**	0.900	8.179	85.139						
**4**	0.653	5.938	91.077						
**5**	0.490	4.456	95.533						
**6**	0.206	1.871	97.404						
**7**	0.155	1.409	98.814						
**8**	0.093	0.845	99.659						
**9**	0.025	0.223	99.881						
**10**	0.008	0.071	99.952						
**11**	0.005	0.048	100.000						
