# Supplementary material for: Elemental pollution and risk assessment of soils and Gundelia tournefortii in a multi-sector industrial zone with a history of agricultural use
Source: PeerJ. 2025 Nov 24;13:e20374. doi: 10.7717/peerj.20374 (PMC12659707; doi:10.7717/peerj.20374)
Supplement: Supplemental Information 24 [file peerj-13-20374-s024.pdf]

Table S24. KMO and Bartlett's Test results for stem samples data set

KMO and Bartlett's Test

**Kaiser-Meyer-Olkin Measure of Sampling Adequacy.**		0.537
**Bartlett's Test of Sphericity**	**Approx. Chi-Square**	135.001
	**df**	55
	**Sig.**	0.000
