# Supplementary material for: Elemental pollution and risk assessment of soils and Gundelia tournefortii in a multi-sector industrial zone with a history of agricultural use
Source: PeerJ. 2025 Nov 24;13:e20374. doi: 10.7717/peerj.20374 (PMC12659707; doi:10.7717/peerj.20374)
Supplement: Supplemental Information 25 [file peerj-13-20374-s025.pdf]

Table S25. Distribution of principal component scores by sampling locations

Soil			Root				Stem		
**Sample#**	**PC1**	**PC2**	**Sample#**	**PC1**	**PC2**	**PC**	**Sample#**	**PC1**	**PC2**
**S1**	-2.20662	1.52561	**RO1**	-1.01803	1.78019	1.14113	**ST1**	-1.13926	0.25624
**S2**	-0.53874	-0.23271	**RO2**	-0.57518	1.34947	0.21546	**ST2**	-0.62196	1.44854
**S3**	-1.11983	-1.35127	**RO3**	1.21825	0.0241	1.54972	**ST3**	-0.83681	1.82343
**S4**	-0.44276	0.64172	**RO4**	1.12141	0.79297	0.18639	**ST4**	0.02504	0.0564
**S5**	0.9152	0.8585	**RO5**	1.78679	0.13953	-0.62678	**ST5**	0.23966	-0.01479
**S6**	-0.68234	-0.99654	**RO6**	-0.52717	-0.96819	1.54335	**ST6**	1.31917	1.20355
**S7**	1.19626	1.29179	**RO7**	1.14471	-0.61963	-0.16027	**ST7**	0.35677	-0.54234
**S8**	0.95769	-0.81485	**RO8**	-1.1042	-0.77356	-0.04761	**ST8**	-0.06793	-1.06781
**S9**	1.11007	-0.50998	**RO9**	-0.88354	-1.68912	-0.14355	**ST9**	0.80088	-0.82979
**S10**	0.22488	-1.27227	**RO10**	-0.02463	-0.15626	-0.69227	**ST10**	2.03465	-0.1726
**S11**	-0.01889	-0.55041	**RO11**	-0.54603	-0.52756	0.12867	**ST11**	-1.40398	-0.5372
**S12**	-0.06688	0.43925	**RO12**	0.26775	-0.40926	-1.31398	**ST12**	0.23129	-0.00562
**S13**	0.67195	0.97117	**RO13**	-0.86015	1.05731	-1.78026	**ST13**	-0.93753	-1.61801
