# Supplementary material for: Elemental pollution and risk assessment of soils and Gundelia tournefortii in a multi-sector industrial zone with a history of agricultural use
Source: PeerJ. 2025 Nov 24;13:e20374. doi: 10.7717/peerj.20374 (PMC12659707; doi:10.7717/peerj.20374)
Supplement: Supplemental Information 26 [file peerj-13-20374-s026.pdf]

Table S26. The Nemerow Composite Pollution Index (NCPI) values for roots

	P_i												
**Elements**	**RO1**	**RO2**	**RO3**	**RO4**	**RO5**	**RO6**	**RO7**	**RO8**	**RO9**	**RO10**	**RO11**	**RO12**	**RO13**
Cd	0.02	0.03	0.01	0.00	0.01	0.05	0.01	0.05	0.05	0.07	0.06	0.05	0.04
Cr	0.05	0.05	0.05	0.05	0.06	0.10	0.10	0.13	0.17	0.07	0.08	0.07	0.06
Cu	**51.46**	**65.05**	**53.18**	**44.71**	**45.61**	**51.33**	**49.33**	**46.94**	**70.77**	**53.04**	**45.21**	**57.43**	**43.18**
Ni	0.02	0.02	0.02	0.02	0.02	0.07	0.05	0.11	0.19	0.11	0.09	0.13	0.04
Pb	**2.41**	**2.33**	**2.00**	**1.59**	**1.80**	**2.25**	**1.84**	**2.52**	**2.45**	**1.81**	**2.72**	**1.76**	**1.68**
Zn	**17.33**	**7.88**	**6.70**	**6.37**	**5.22**	**9.92**	**3.43**	**2.42**	**3.06**	**3.49**	**2.57**	**3.35**	**2.94**
Fe	**8.55**	**8.69**	**1.36**	**4.15**	**6.38**	**3.74**	**5.85**	**4.76**	**15.55**	**2.58**	**2.02**	**2.94**	**16.69**
Mn	**6.15**	**10.94**	**6.28**	**5.63**	**6.46**	**5.33**	**7.16**	**5.53**	**9.15**	**8.28**	**4.96**	**5.99**	**5.51**
P_{i-mean}	**10.75**	**11.87**	**8.70**	**7.82**	**8.19**	**9.10**	**8.47**	**7.81**	**12.67**	**8.68**	**7.21**	**8.97**	**8.77**
P_{i-max}	**51.46**	**10.94**	**6.28**	**5.63**	**6.46**	**5.33**	**7.16**	**5.53**	**15.55**	**8.28**	**4.96**	**5.99**	**16.69**
**NCPI**	**26.29**	**8.07**	**5.36**	**4.82**	**5.22**	**5.27**	**5.55**	**4.78**	**10.03**	**6.00**	**4.38**	**5.39**	**9.43**

If the P_i value is > 1, the food sample is evaluated as contaminated.

NCPI<1.0: uncontaminated food, 1.0≤NCPI<2.5: lightly contaminated food, 2.5≤NCPI<7: moderately contaminated food and NCPI≥7: heavily contaminated food
