# Supplementary material for: Elemental pollution and risk assessment of soils and Gundelia tournefortii in a multi-sector industrial zone with a history of agricultural use
Source: PeerJ. 2025 Nov 24;13:e20374. doi: 10.7717/peerj.20374 (PMC12659707; doi:10.7717/peerj.20374)
Supplement: Supplemental Information 27 [file peerj-13-20374-s027.pdf]

Table S27. The Nemerow Composite Pollution Index (NCPI) values for stems

	P_i												
**Elements**	**ST1**	**ST2**	**ST3**	**ST4**	**ST5**	**ST6**	**ST7**	**ST8**	**ST9**	**ST10**	**ST11**	**ST12**	**ST13**
Cd	0.43	0.44	0.58	0.14	0.14	0.15	0.14	0.05	0.13	0.07	0.10	0.35	0.11
Cr	0.10	0.08	0.08	0.10	0.09	0.04	0.04	0.04	0.03	0.03	0.10	0.03	0.10
Cu	**17.18**	**20.36**	**14.91**	**22.24**	**24.50**	**21.01**	**16.91**	**11.08**	**18.57**	**22.27**	**11.93**	**14.64**	**13.32**
Ni	0.09	0.11	0.09	0.16	0.08	0.05	0.08	0.11	0.07	0.04	0.07	0.04	0.15
Pb	**2.17**	**1.71**	**1.84**	**1.28**	**1.71**	0.61	0.74	0.60	0.53	0.40	**1.55**	0.77	0.63
Zn	**3.87**	**3.00**	**2.12**	**2.83**	**3.10**	**3.62**	**0.81**	**0.91**	**0.78**	**1.00**	**1.43**	**1.36**	**0.65**
Fe	**1.85**	**3.84**	**1.39**	**4.08**	**3.96**	**1.06**	**2.08**	**1.12**	**4.28**	**2.60**	**1.33**	**1.08**	**6.52**
Mn	**2.43**	**2.30**	**1.85**	**3.16**	**3.03**	**2.57**	**2.12**	**1.53**	**2.21**	**2.69**	**1.53**	**1.87**	**1.93**
Pi mean	**3.52**	**3.98**	**2.86**	**4.25**	**4.58**	**3.64**	**2.86**	**1.93**	**3.32**	**3.64**	**2.26**	**2.52**	**2.93**
Pi max	**17.18**	**3.84**	**1.85**	**4.08**	**3.96**	**2.57**	**2.12**	**1.53**	**4.28**	**2.69**	**1.53**	**1.87**	**6.52**
**NCPI**	**8.77**	**2.76**	**1.70**	**2.95**	**3.03**	**2.23**	**1.78**	**1.23**	**2.71**	**2.26**	**1.36**	**1.57**	**3.58**

If the P_i value is > 1, the food sample is evaluated as contaminated.

NCPI<1.0: uncontaminated food, 1.0≤NCPI<2.5: lightly contaminated food, 2.5≤NCPI<7: moderately contaminated food and NCPI≥7: heavily contaminated food
