# Supplementary material for: Elemental pollution and risk assessment of soils and Gundelia tournefortii in a multi-sector industrial zone with a history of agricultural use
Source: PeerJ. 2025 Nov 24;13:e20374. doi: 10.7717/peerj.20374 (PMC12659707; doi:10.7717/peerj.20374)
Supplement: Supplemental Information 28 [file peerj-13-20374-s028.pdf]

Table S28. Estimated daily intake (EDI) of heavy metals in root samples for adults

Elements mg/kg bw.day	RO1	RO2	RO3	RO4	RO5	RO6	RO7	RO8	RO9	RO10	RO11	RO12	RO13	$TUIL_{man}$	$TUIL_{woman}$
**Cd**	0.0000	0.0000	0.0000	0.0000	0.0000	0.0000	0.0000	0.0000	0.0000	0.0000	0.0000	0.0000	0.0000	0.0009	0.0009
**Cr**	0.0000	0.0000	0.0000	0.0000	0.0000	0.0000	0.0000	0.0001	0.0001	0.0000	0.0000	0.0000	0.0000	0.0005	0.0003
**Cu**	0.09	0.12	0.09	0.08	0.08	0.09	0.09	0.08	0.13	0.09	0.08	0.10	0.08	0.1357	0.1357
**Ni**	0.0000	0.0000	0.0000	0.0000	0.0000	0.0000	0.0000	0.0000	0.0000	0.0000	0.0000	0.0000	0.0000	0.0136	0.0136
**Pb**	0.0000	0.0000	0.0000	0.0000	0.0000	0.0000	0.0000	0.0000	0.0000	0.0000	0.0000	0.0000	0.0000	0.0036	0.0036
**Zn**	0.06	0.03	0.02	0.02	0.02	0.04	0.01	0.01	0.01	0.01	0.01	0.01	0.01	0.5427	0.5427
**Fe**	0.07	0.07	0.01	0.03	0.05	0.03	0.05	0.04	0.12	0.02	0.02	0.02	0.13	0.6106	0.6106
**Mn**	0.02	0.03	0.02	0.02	0.02	0.02	0.02	0.02	0.03	0.02	0.01	0.02	0.02	0.1493	0.1493

TUIL: Tolerable Upper Intake Level
