# Supplementary material for: Elemental pollution and risk assessment of soils and Gundelia tournefortii in a multi-sector industrial zone with a history of agricultural use
Source: PeerJ. 2025 Nov 24;13:e20374. doi: 10.7717/peerj.20374 (PMC12659707; doi:10.7717/peerj.20374)
Supplement: Supplemental Information 29 [file peerj-13-20374-s029.pdf]

Table S29. Estimated daily intake (EDI) of heavy metals in stem samples for adults

Elements mg/kg bw.day	ST1	ST2	ST3	ST4	ST5	ST6	ST7	ST8	ST9	ST10	ST11	ST12	ST13	$TUIL_{man}$	$TUIL_{woman}$
**Cd**	0.0000	0.0000	0.0000	0.0000	0.0000	0.0000	0.0000	0.0000	0.0000	0.0000	0.0000	0.0000	0.0000	0.0009	0.0009
**Cr**	0.0000	0.0000	0.0000	0.0000	0.0000	0.0000	0.0000	0.0000	0.0000	0.0000	0.0000	0.0000	0.0000	0.0005	0.0003
**Cu**	0.03	0.04	0.03	0.04	0.04	0.04	0.03	0.02	0.03	0.04	0.02	0.03	0.02	0.1357	0.1357
**Ni**	0.0000	0.0000	0.0000	0.0000	0.0000	0.0000	0.0000	0.0000	0.0000	0.0000	0.0000	0.0000	0.0000	0.0136	0.0136
**Pb**	0.0000	0.0000	0.0000	0.0000	0.0000	0.0000	0.0000	0.0000	0.0000	0.0000	0.0000	0.0000	0.0000	0.0036	0.0036
**Zn**	0.014	0.011	0.008	0.010	0.011	0.013	0.003	0.003	0.003	0.004	0.005	0.005	0.002	0.5427	0.5427
**Fe**	0.01	0.03	0.01	0.03	0.03	0.01	0.02	0.01	0.03	0.02	0.01	0.01	0.05	0.6106	0.6106
**Mn**	0.01	0.01	0.01	0.01	0.01	0.01	0.01	0.00	0.01	0.01	0.00	0.01	0.01	0.1493	0.1493

TUIL: Tolerable Upper Intake Level
