# Supplementary material for: Elemental pollution and risk assessment of soils and Gundelia tournefortii in a multi-sector industrial zone with a history of agricultural use
Source: PeerJ. 2025 Nov 24;13:e20374. doi: 10.7717/peerj.20374 (PMC12659707; doi:10.7717/peerj.20374)
Supplement: Supplemental Information 30 [file peerj-13-20374-s030.pdf]

Table S30. Estimated daily intake (EDI) of heavy metals in root samples for children

Elements mg/kg bw.day	RO1	RO2	RO3	RO4	RO5	RO6	RO7	RO8	RO9	RO10	RO11	RO12	RO13	$TUIL_{child}$
**Cd**	0.0000	0.0000	0.0000	0.0000	0.0000	0.0000	0.0000	0.0000	0.0000	0.0000	0.0000	0.0000	0.0000	0.0009
**Cr**	0.0007	0.0008	0.0007	0.0008	0.0009	**0.0015**	**0.0015**	**0.0020**	**0.0027**	**0.0012**	**0.0013**	**0.0010**	**0.0009**	0.0009
**Cu**	**3.47**	**4.38**	**3.58**	**3.01**	**3.07**	**3.46**	**3.32**	**3.16**	**4.77**	**3.58**	**3.05**	**3.87**	**2.91**	0.188
**Ni**	0.0002	0.0002	0.0002	0.0002	0.0002	0.0007	0.0005	0.0011	0.0019	0.0012	0.0009	0.0013	0.0004	0.019
**Pb**	0.0016	0.0016	0.0013	0.0011	0.0012	0.0015	0.0012	0.0017	0.0017	0.0012	0.0018	0.0012	0.0011	0.0036
**Zn**	**2.34**	**1.06**	**0.90**	**0.86**	0.70	**1.34**	0.46	0.33	0.41	0.47	0.35	0.45	0.40	0.75
**Fe**	**2.59**	**2.63**	0.41	1.26	1.93	1.14	1.77	1.44	**4.72**	0.78	0.61	0.89	**5.06**	2.5
**Mn**	**0.69**	**1.23**	**0.70**	**0.63**	**0.72**	**0.60**	**0.80**	**0.62**	**1.02**	**0.93**	**0.56**	**0.67**	**0.62**	0.188

TUIL: Tolerable Upper Intake Level

Bolded numbers: The values exceeding Tolerable Upper Intake Level (TUIL).
