# Supplementary material for: Elemental pollution and risk assessment of soils and Gundelia tournefortii in a multi-sector industrial zone with a history of agricultural use
Source: PeerJ. 2025 Nov 24;13:e20374. doi: 10.7717/peerj.20374 (PMC12659707; doi:10.7717/peerj.20374)
Supplement: Supplemental Information 31 [file peerj-13-20374-s031.pdf]

Table S31. Estimated daily intake (EDI) of heavy metals in stem samples for children

Elements mg/kg bw.day	ST1	ST2	ST3	ST4	ST5	ST6	ST7	ST8	ST9	ST10	ST11	ST12	ST13	$TUIL_{child}$
**Cd**	0.0003	0.0003	0.0004	0.0001	0.0001	0.0001	0.0001	0.0000	0.0001	0.0000	0.0001	0.0002	0.0001	0.0009
**Cr**	**0.0015**	**0.0013**	**0.0013**	**0.0016**	**0.0015**	0.0006	0.0006	0.0006	0.0005	0.0005	**0.0016**	0.0005	**0.0016**	0.0009
**Cu**	**1.16**	**1.37**	**1.00**	**1.50**	**1.65**	**1.42**	**1.14**	**0.75**	**1.25**	**1.50**	**0.80**	**0.99**	**0.90**	0.188
**Ni**	0.0010	0.0011	0.0009	0.0016	0.0008	0.0005	0.0008	0.0011	0.0007	0.0004	0.0007	0.0004	0.0015	0.019
**Pb**	0.0015	0.0012	0.0012	0.0009	0.0011	0.0004	0.0005	0.0004	0.0004	0.0003	0.0010	0.0005	0.0004	0.0036
**Zn**	0.52	0.40	0.29	0.38	0.42	0.49	0.11	0.12	0.10	0.14	0.19	0.18	0.09	0.75
**Fe**	0.56	1.16	0.42	1.24	1.20	0.32	0.63	0.34	1.30	0.79	0.40	0.33	1.98	2.5
**Mn**	**0.27**	**0.26**	**0.21**	**0.35**	**0.34**	**0.29**	**0.24**	0.17	**0.25**	**0.30**	0.17	**0.21**	**0.22**	0.188

TUIL: Tolerable Upper Intake Level

Bolded numbers: The values exceeding Tolerable Upper Intake Level (TUIL).
