# Supplementary material for: Elemental pollution and risk assessment of soils and Gundelia tournefortii in a multi-sector industrial zone with a history of agricultural use
Source: PeerJ. 2025 Nov 24;13:e20374. doi: 10.7717/peerj.20374 (PMC12659707; doi:10.7717/peerj.20374)
Supplement: Supplemental Information 32 [file peerj-13-20374-s032.pdf]

**Table S32.** Hazard quotient (HQ) and hazard index (HI) of heavy metals in root samples for adults

| | HQ | | | | | | | | | | | | |
|---|---|---|---|---|---|---|---|---|---|---|---|---|---|
| **Elements** | **RO1** | **RO2** | **RO3** | **RO4** | **RO5** | **RO6** | **RO7** | **RO8** | **RO9** | **RO10** | **RO11** | **RO12** | **RO13** |
| **Cd** | 0.0003 | 0.001 | 0.0002 | 0.0001 | 0.0001 | 0.001 | 0.0001 | 0.001 | 0.001 | 0.001 | 0.001 | 0.001 | 0.001 |
| **Cr** | 0.02 | 0.02 | 0.02 | 0.02 | 0.03 | 0.05 | 0.05 | 0.06 | 0.08 | 0.03 | 0.04 | 0.03 | 0.03 |
| **Cu** | **2.30** | **2.90** | **2.37** | **1.99** | **2.03** | **2.29** | **2.20** | **2.09** | **3.16** | **2.37** | **2.02** | **2.56** | **1.93** |
| **Ni** | 0.0002 | 0.0003 | 0.0002 | 0.0003 | 0.0003 | 0.001 | 0.001 | 0.001 | 0.002 | 0.002 | 0.001 | 0.002 | 0.001 |
| **Pb** | 0.03 | 0.03 | 0.03 | 0.02 | 0.02 | 0.03 | 0.02 | 0.03 | 0.03 | 0.02 | 0.03 | 0.02 | 0.02 |
| **Zn** | 0.21 | 0.09 | 0.08 | 0.08 | 0.06 | 0.12 | 0.04 | 0.03 | 0.04 | 0.04 | 0.03 | 0.04 | 0.04 |
| **Fe** | 0.10 | 0.10 | 0.02 | 0.05 | 0.07 | 0.04 | 0.07 | 0.05 | 0.18 | 0.03 | 0.02 | 0.03 | 0.19 |
| **Mn** | 0.13 | 0.23 | 0.13 | 0.12 | 0.14 | 0.11 | 0.15 | 0.12 | 0.19 | 0.18 | 0.11 | 0.13 | 0.12 |
| **HI** | **2.78** | **3.38** | **2.65** | **2.28** | **2.36** | **2.64** | **2.53** | **2.39** | **3.68** | **2.67** | **2.25** | **2.82** | **2.32** |

HI ≥ 1 → Non-cancer risk is possible

HQ ≥ 1 → Potential health concern
