# Supplementary material for: Elemental pollution and risk assessment of soils and Gundelia tournefortii in a multi-sector industrial zone with a history of agricultural use
Source: PeerJ. 2025 Nov 24;13:e20374. doi: 10.7717/peerj.20374 (PMC12659707; doi:10.7717/peerj.20374)
Supplement: Supplemental Information 33 [file peerj-13-20374-s033.pdf]

Table S33. Hazard quotient (HQ) and hazard index (HI) of heavy metals in root samples for children

	HQ												
**Elements**	**RO1**	**RO2**	**RO3**	**RO4**	**RO5**	**RO6**	**RO7**	**RO8**	**RO9**	**RO10**	**RO11**	**RO12**	**RO13**
**Cd**	0.01	0.02	0.01	0.003	0.004	0.03	0.01	0.03	0.04	0.04	0.04	0.04	0.03
**Cr**	0.82	0.92	0.81	0.85	0.98	**1.71**	**1.71**	**2.18**	**2.99**	**1.28**	**1.44**	**1.14**	**1.04**
**Cu**	**86.71**	**109.61**	**89.61**	**75.33**	**76.85**	**86.49**	**83.12**	**79.09**	**119.24**	**89.38**	**76.17**	**96.77**	**72.76**
**Ni**	0.01	0.01	0.01	0.01	0.01	0.04	0.02	0.06	0.09	0.06	0.04	0.06	0.02
**Pb**	**1.16**	**1.12**	0.96	0.76	0.87	**1.08**	0.89	**1.22**	**1.18**	0.87	**1.31**	0.85	0.81
**Zn**	**7.78**	**3.54**	**3.01**	**2.86**	**2.34**	**4.46**	**1.54**	**1.09**	**1.37**	**1.57**	**1.15**	**1.51**	**1.32**
**Fe**	**3.70**	**3.76**	0.59	**1.80**	**2.76**	**1.62**	**2.53**	**2.06**	**6.74**	**1.12**	0.88	**1.27**	**7.23**
**Mn**	**4.92**	**8.75**	**5.02**	**4.50**	**5.16**	**4.26**	**5.73**	**4.42**	**7.32**	**6.62**	**3.97**	**4.79**	**4.41**
**HI**	**105.12**	**127.73**	**100.01**	**86.12**	**88.99**	**99.70**	**95.54**	**90.15**	**138.97**	**100.94**	**85.00**	**106.43**	**87.63**

HI ≥ 1 → Non-cancer risk is possible

HQ ≥ 1 → Potential health concern
