# Supplementary material for: Elemental pollution and risk assessment of soils and Gundelia tournefortii in a multi-sector industrial zone with a history of agricultural use
Source: PeerJ. 2025 Nov 24;13:e20374. doi: 10.7717/peerj.20374 (PMC12659707; doi:10.7717/peerj.20374)
Supplement: Supplemental Information 34 [file peerj-13-20374-s034.pdf]

Table S34. Hazard quotient (HQ) and hazard index (HI) of heavy metals in stem samples for adults

	HQ												
**Elements**	**ST1**	**ST2**	**ST3**	**ST4**	**ST5**	**ST6**	**ST7**	**ST8**	**ST9**	**ST10**	**ST11**	**ST12**	**ST13**
**Cd**	0.01	0.01	0.01	0.003	0.002	0.003	0.002	0.001	0.002	0.001	0.002	0.01	0.002
**Cr**	0.01	0.01	0.01	0.01	0.01	0.01	0.01	0.01	0.005	0.004	0.01	0.004	0.01
**Cu**	0.77	0.91	0.67	0.99	**1.09**	0.94	0.75	0.49	0.83	0.99	0.53	0.65	0.59
**Ni**	0.001	0.001	0.001	0.002	0.001	0.001	0.001	0.002	0.001	0.0005	0.001	0.001	0.002
**Pb**	0.01	0.01	0.01	0.01	0.01	0.003	0.004	0.003	0.003	0.002	0.01	0.004	0.003
**Zn**	0.05	0.04	0.03	0.03	0.04	0.04	0.01	0.01	0.01	0.01	0.02	0.02	0.01
**Fe**	0.02	0.04	0.02	0.05	0.05	0.01	0.02	0.01	0.05	0.03	0.02	0.01	0.07
**Mn**	0.05	0.05	0.04	0.07	0.06	0.05	0.04	0.03	0.05	0.06	0.03	0.04	0.04
**HI**	0.92	**1.07**	0.78	**1.16**	**1.26**	**1.06**	0.85	0.56	0.94	**1.10**	0.62	0.74	0.74

HI ≥ 1 → Non-cancer risk is possible

HQ ≥ 1 → Potential health concern
