# Supplementary material for: Elemental pollution and risk assessment of soils and Gundelia tournefortii in a multi-sector industrial zone with a history of agricultural use
Source: PeerJ. 2025 Nov 24;13:e20374. doi: 10.7717/peerj.20374 (PMC12659707; doi:10.7717/peerj.20374)
Supplement: Supplemental Information 35 [file peerj-13-20374-s035.pdf]

Table S35. Hazard quotient (HQ) and hazard index (HI) of heavy metals in stem samples for children

	HQ												
**Elements**	**ST1**	**ST2**	**ST3**	**ST4**	**ST5**	**ST6**	**ST7**	**ST8**	**ST9**	**ST10**	**ST11**	**ST12**	**ST13**
**Cd**	0.29	0.30	0.39	0.10	0.09	0.10	0.09	0.04	0.09	0.05	0.07	0.23	0.07
**Cr**	0.50	0.43	0.42	0.52	0.49	0.19	0.20	0.20	0.17	0.16	0.52	0.17	0.52
**Cu**	**28.95**	**34.31**	**25.12**	**37.48**	**41.27**	**35.40**	**28.50**	**18.66**	**31.29**	**37.53**	**20.11**	**24.67**	**22.44**
**Ni**	0.05	0.06	0.05	0.08	0.04	0.02	0.04	0.06	0.03	0.02	0.03	0.02	0.08
**Pb**	0.42	0.33	0.35	0.25	0.33	0.12	0.14	0.12	0.10	0.08	0.30	0.15	0.12
**Zn**	**1.74**	**1.35**	0.95	**1.27**	**1.39**	**1.63**	0.36	0.41	0.35	0.45	0.64	0.61	0.29
**Fe**	0.80	**1.66**	0.60	**1.77**	**1.72**	0.46	0.90	0.49	**1.85**	**1.13**	0.57	0.47	**2.83**
**Mn**	**1.94**	**1.84**	**1.48**	**2.53**	**2.43**	**2.06**	**1.69**	**1.23**	**1.77**	**2.15**	**1.22**	**1.49**	**1.54**
**HI**	**34.69**	**40.27**	**29.36**	**43.99**	**47.76**	**39.98**	**31.93**	**21.20**	**35.65**	**41.56**	**23.46**	**27.81**	**27.90**

HI ≥ 1 → Non-cancer risk is possible

HQ ≥ 1 → Potential health concern
