# Supplementary material for: Elemental pollution and risk assessment of soils and Gundelia tournefortii in a multi-sector industrial zone with a history of agricultural use
Source: PeerJ. 2025 Nov 24;13:e20374. doi: 10.7717/peerj.20374 (PMC12659707; doi:10.7717/peerj.20374)
Supplement: Supplemental Information 36 [file peerj-13-20374-s036.pdf]

Table S36. Lifetime Cancer Risk (CR) of heavy metals in root samples for adults

	CR												
**Elements**	**RO1**	**RO2**	**RO3**	**RO4**	**RO5**	**RO6**	**RO7**	**RO8**	**RO9**	**RO10**	**RO11**	**RO12**	**RO13**
**Cd**	5.06E-06	7.52E-06	2.33E-06	1.19E-06	1.71E-06	1.38E-05	2.01E-06	1.38E-05	1.42E-05	1.74E-05	1.72E-05	1.46E-05	1.20E-05
**Cr**	5.30E-06	5.90E-06	5.19E-06	5.48E-06	6.33E-06	1.10E-05	1.10E-05	1.40E-05	1.92E-05	8.25E-06	9.25E-06	7.35E-06	6.72E-06
**Ni**	4.25E-07	5.26E-07	3.92E-07	4.60E-07	4.88E-07	1.74E-06	1.13E-06	2.63E-06	4.49E-06	2.76E-06	2.07E-06	3.06E-06	9.36E-07
**Pb**	3.65E-07	3.53E-07	3.04E-07	2.41E-07	2.74E-07	3.41E-07	2.79E-07	3.83E-07	3.71E-07	2.74E-07	4.13E-07	2.66E-07	2.55E-07
**Total Risk**	1.12E-05	1.43E-05	8.22E-06	7.38E-06	8.81E-06	2.68E-05	1.44E-05	3.08E-05	3.84E-05	2.87E-05	2.90E-05	2.53E-05	1.99E-05

$< 1\times10^{-6}$: Negligible risk, 1×10^{-6} to 1×10^{-4} : Acceptable risk range, **$> 1\times10^{-4}$: Unacceptable/high risk**
