# Supplementary material for: Elemental pollution and risk assessment of soils and Gundelia tournefortii in a multi-sector industrial zone with a history of agricultural use
Source: PeerJ. 2025 Nov 24;13:e20374. doi: 10.7717/peerj.20374 (PMC12659707; doi:10.7717/peerj.20374)
Supplement: Supplemental Information 37 [file peerj-13-20374-s037.pdf]

Table S37. Lifetime Cancer Risk (CR) of heavy metals in root samples for children

	CR												
**Elements**	**RO1**	**RO2**	**RO3**	**RO4**	**RO5**	**RO6**	**RO7**	**RO8**	**RO9**	**RO10**	**RO11**	**RO12**	**RO13**
**Cd**	**1.91E-04**	**2.84E-04**	8.81E-05	4.51E-05	6.48E-05	**5.20E-04**	7.60E-05	**5.20E-04**	**5.38E-04**	**6.58E-04**	**6.50E-04**	**5.52E-04**	**4.52E-04**
**Cr**	**2.00E-04**	**2.23E-04**	**1.96E-04**	**2.07E-04**	**2.39E-04**	**4.15E-04**	**4.15E-04**	**5.30E-04**	**7.27E-04**	**3.12E-04**	**3.49E-04**	**2.78E-04**	**2.54E-04**
**Ni**	1.61E-05	1.98E-05	1.48E-05	1.74E-05	1.84E-05	6.59E-05	4.27E-05	9.95E-05	**1.70E-04**	**1.04E-04**	7.81E-05	**1.16E-04**	3.53E-05
**Pb**	1.38E-05	1.33E-05	1.15E-05	9.09E-06	1.03E-05	1.29E-05	1.06E-05	1.45E-05	1.40E-05	1.03E-05	1.56E-05	1.01E-05	9.64E-06
**Total Risk**	**4.21E-04**	**5.40E-04**	**3.10E-04**	**2.79E-04**	**3.33E-04**	**1.01E-03**	**5.44E-04**	**1.16E-03**	**1.45E-03**	**1.08E-03**	**1.09E-03**	**9.56E-04**	**7.51E-04**

$< 1\times10^{-6}$: Negligible risk, 1×10^{-6} to 1×10^{-4} : Acceptable risk range, **$> 1\times10^{-4}$: Unacceptable/high risk**
