# Supplementary material for: Elemental pollution and risk assessment of soils and Gundelia tournefortii in a multi-sector industrial zone with a history of agricultural use
Source: PeerJ. 2025 Nov 24;13:e20374. doi: 10.7717/peerj.20374 (PMC12659707; doi:10.7717/peerj.20374)
Supplement: Supplemental Information 38 [file peerj-13-20374-s038.pdf]

Table S38. Lifetime Cancer Risk (CR) of heavy metals in stem samples for adults

	CR												
**Elements**	**ST1**	**ST2**	**ST3**	**ST4**	**ST5**	**ST6**	**ST7**	**ST8**	**ST9**	**ST10**	**ST11**	**ST12**	**ST13**
**Cd**	**1.15E-04**	**1.19E-04**	**1.56E-04**	3.83E-05	3.70E-05	4.13E-05	3.67E-05	1.47E-05	3.57E-05	1.80E-05	2.68E-05	9.33E-05	2.94E-05
**Cr**	1.07E-05	9.23E-06	8.94E-06	1.11E-05	1.05E-05	4.12E-06	4.33E-06	4.32E-06	3.66E-06	3.46E-06	1.11E-05	3.58E-06	1.12E-05
**Ni**	2.28E-06	2.64E-06	2.24E-06	3.86E-06	1.96E-06	1.11E-06	1.84E-06	2.73E-06	1.63E-06	8.56E-07	1.61E-06	9.46E-07	3.61E-06
**Pb**	3.30E-07	2.60E-07	2.79E-07	1.95E-07	2.59E-07	9.21E-08	1.12E-07	9.10E-08	8.00E-08	6.03E-08	2.36E-07	1.17E-07	9.56E-08
**Total Risk**	**1.28E-04**	**1.31E-04**	**1.68E-04**	5.35E-05	4.97E-05	4.66E-05	4.30E-05	2.18E-05	4.11E-05	2.23E-05	3.97E-05	9.79E-05	4.44E-05

$< 1\times10^{-6}$: Negligible risk, 1×10^{-6} to 1×10^{-4} : Acceptable risk range, **$> 1\times10^{-4}$: Unacceptable/high risk**
