# Supplementary material for: Elemental pollution and risk assessment of soils and Gundelia tournefortii in a multi-sector industrial zone with a history of agricultural use
Source: PeerJ. 2025 Nov 24;13:e20374. doi: 10.7717/peerj.20374 (PMC12659707; doi:10.7717/peerj.20374)
Supplement: Supplemental Information 39 [file peerj-13-20374-s039.pdf]

Table S39. Lifetime Cancer Risk (CR) of heavy metals in stem samples for children

	CR												
**Elements**	**ST1**	**ST2**	**ST3**	**ST4**	**ST5**	**ST6**	**ST7**	**ST8**	**ST9**	**ST10**	**ST11**	**ST12**	**ST13**
**Cd**	**4.35E-03**	**1.82E-03**	**2.40E-03**	**5.89E-04**	**5.68E-04**	**6.34E-04**	**5.64E-04**	**2.26E-04**	**5.49E-04**	**2.76E-04**	**4.12E-04**	**1.43E-03**	**4.52E-04**
**Cr**	**4.03E-04**	**3.48E-04**	**3.38E-04**	**4.21E-04**	**3.96E-04**	**1.56E-04**	**1.64E-04**	**1.63E-04**	**1.38E-04**	**1.31E-04**	**4.19E-04**	**1.35E-04**	**4.24E-04**
**Ni**	8.60E-05	9.96E-05	8.45E-05	**1.46E-04**	7.42E-05	4.17E-05	6.95E-05	**1.03E-04**	6.17E-05	3.23E-05	6.06E-05	3.57E-05	**1.36E-04**
**Pb**	1.24E-05	9.80E-06	1.05E-05	7.36E-06	9.77E-06	3.48E-06	4.25E-06	3.44E-06	3.02E-06	2.28E-06	8.91E-06	4.43E-06	3.61E-06
**Total Risk**	**4.85E-03**	**2.28E-03**	**2.83E-03**	**1.16E-03**	**1.05E-03**	**8.35E-04**	**8.02E-04**	**4.95E-04**	**7.52E-04**	**4.41E-04**	**9.00E-04**	**1.61E-03**	**1.02E-03**

$< 1\times10^{-6}$: Negligible risk, 1×10^{-6} to 1×10^{-4} : Acceptable risk range, **$> 1\times10^{-4}$: Unacceptable/high risk**
