# Supplementary figures and images for: Elemental pollution and risk assessment of soils and Gundelia tournefortii in a multi-sector industrial zone with a history of agricultural use
Source: PeerJ. 2025 Nov 24;13:e20374. doi: 10.7717/peerj.20374 (PMC12659707; doi:10.7717/peerj.20374)

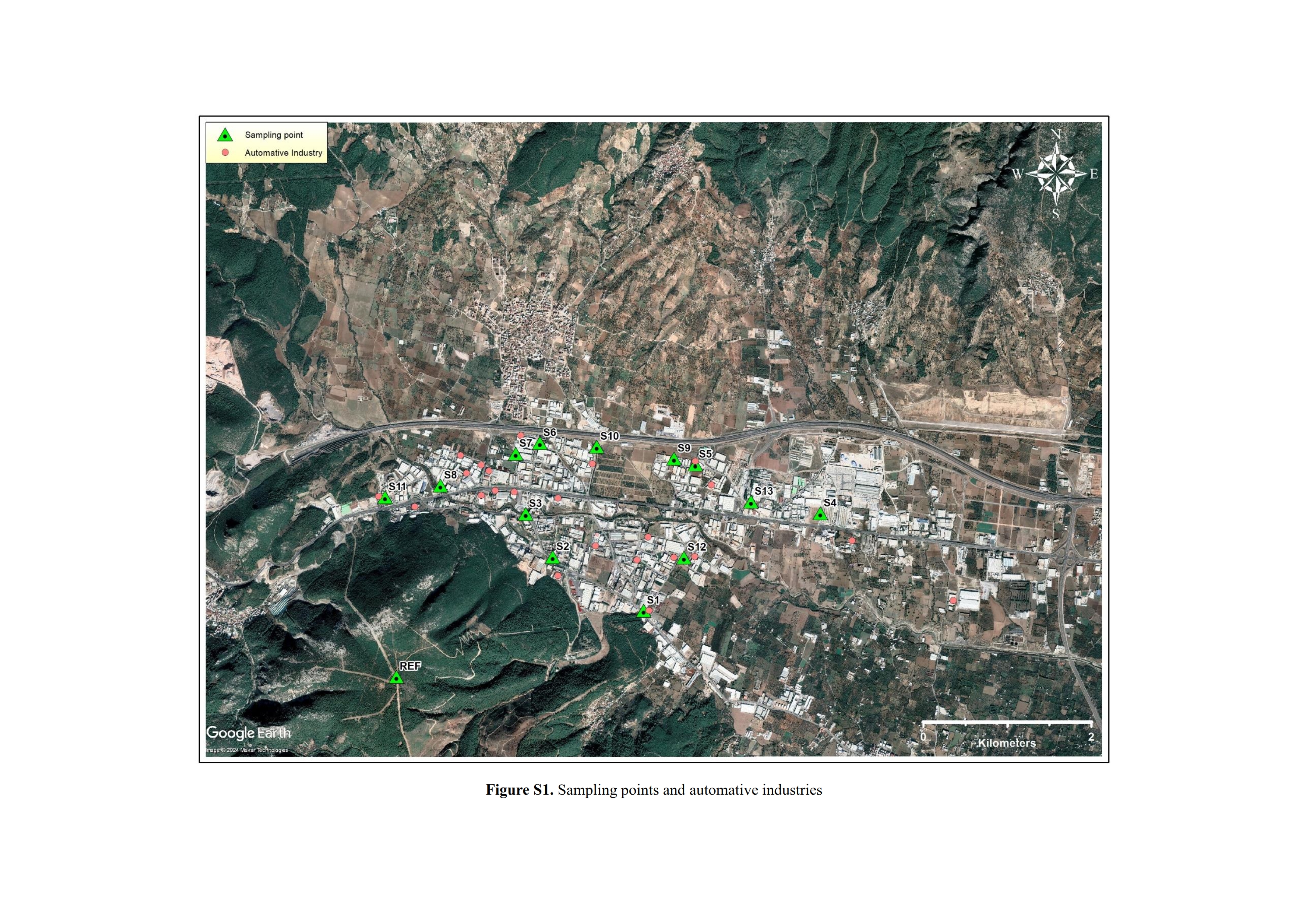

Supplement: Supplemental Information 40 [file peerj-13-20374-s040.jpg]

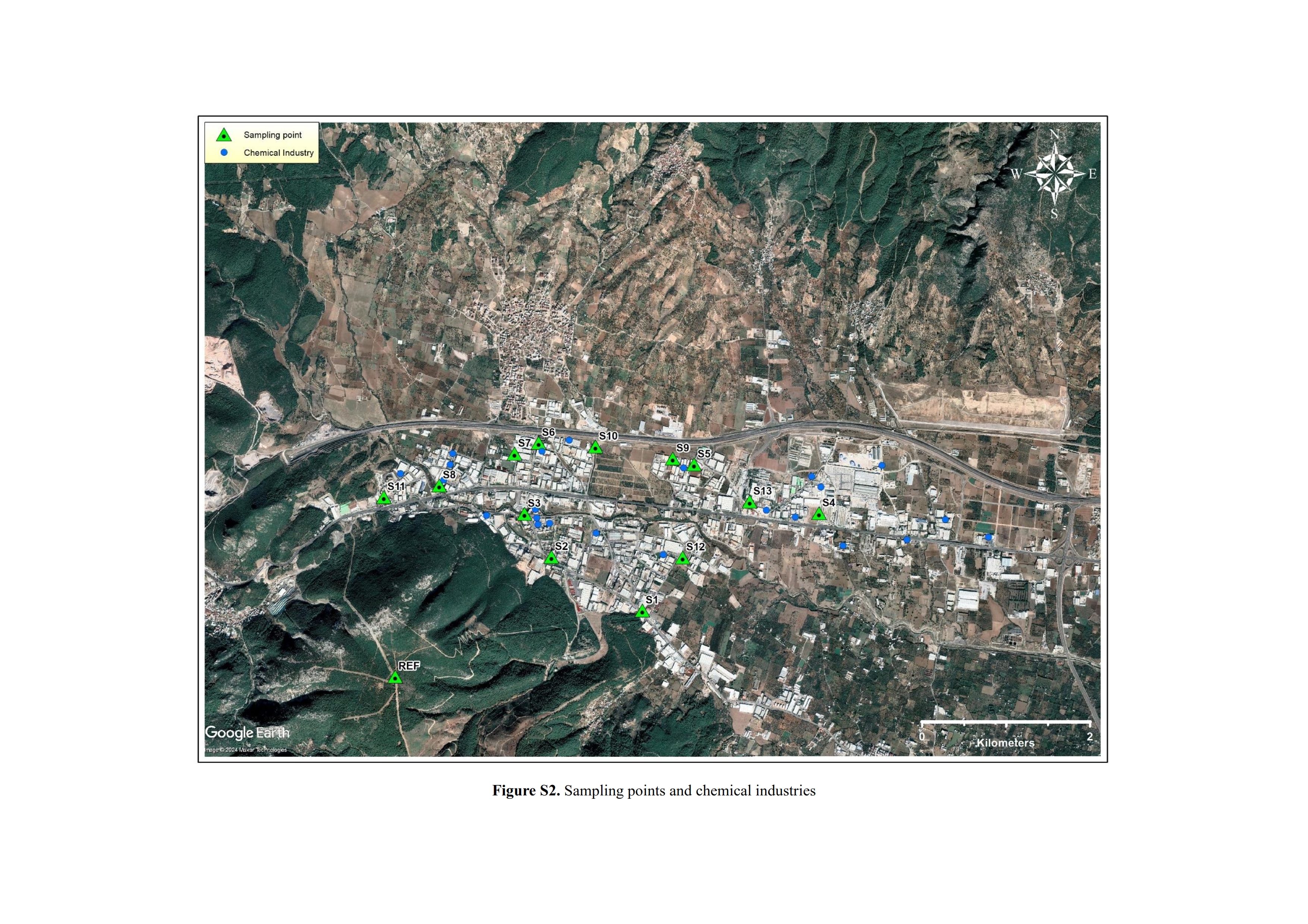

Supplement: Supplemental Information 41 [file peerj-13-20374-s041.jpg]

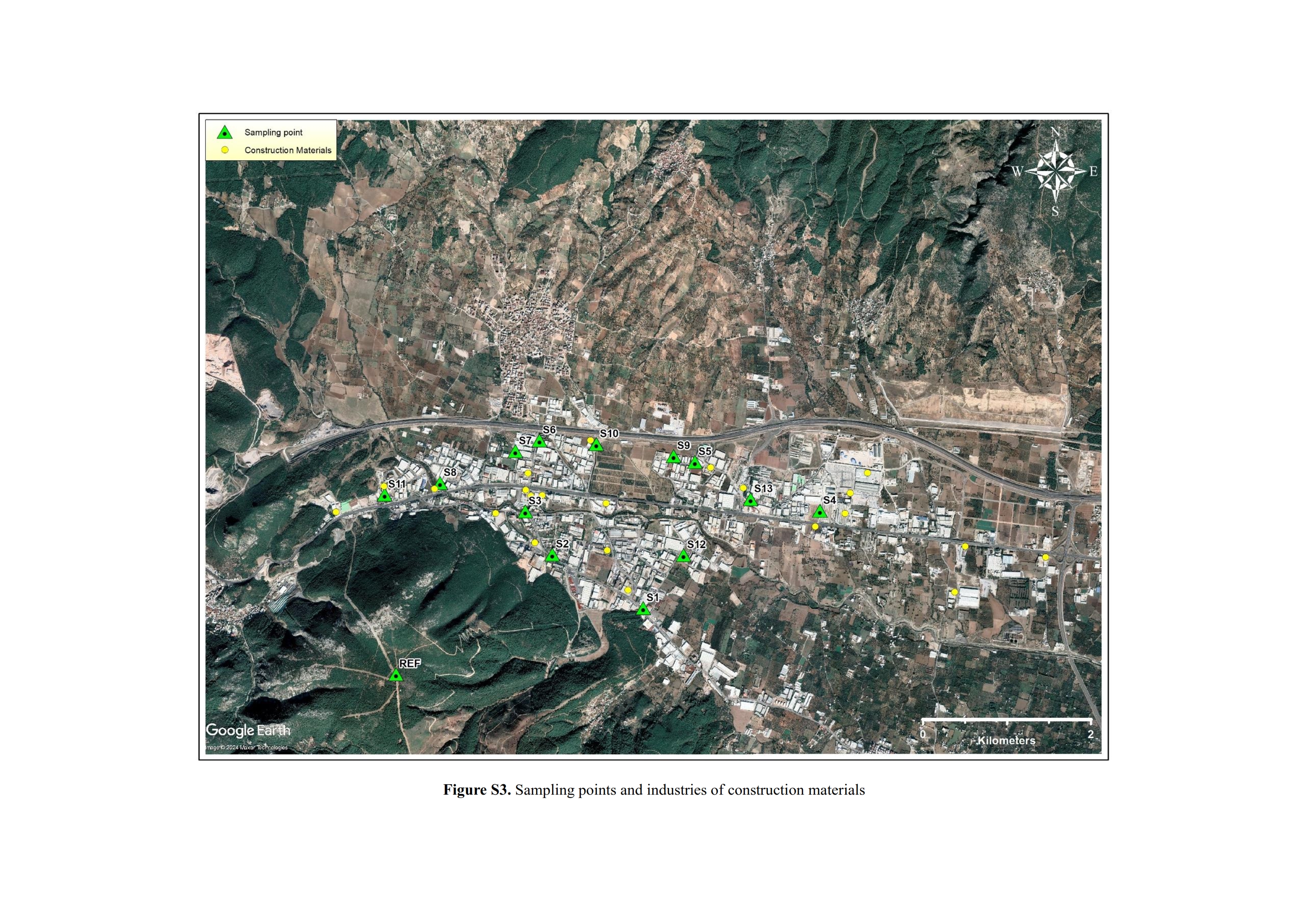

Supplement: Supplemental Information 42 [file peerj-13-20374-s042.jpg]

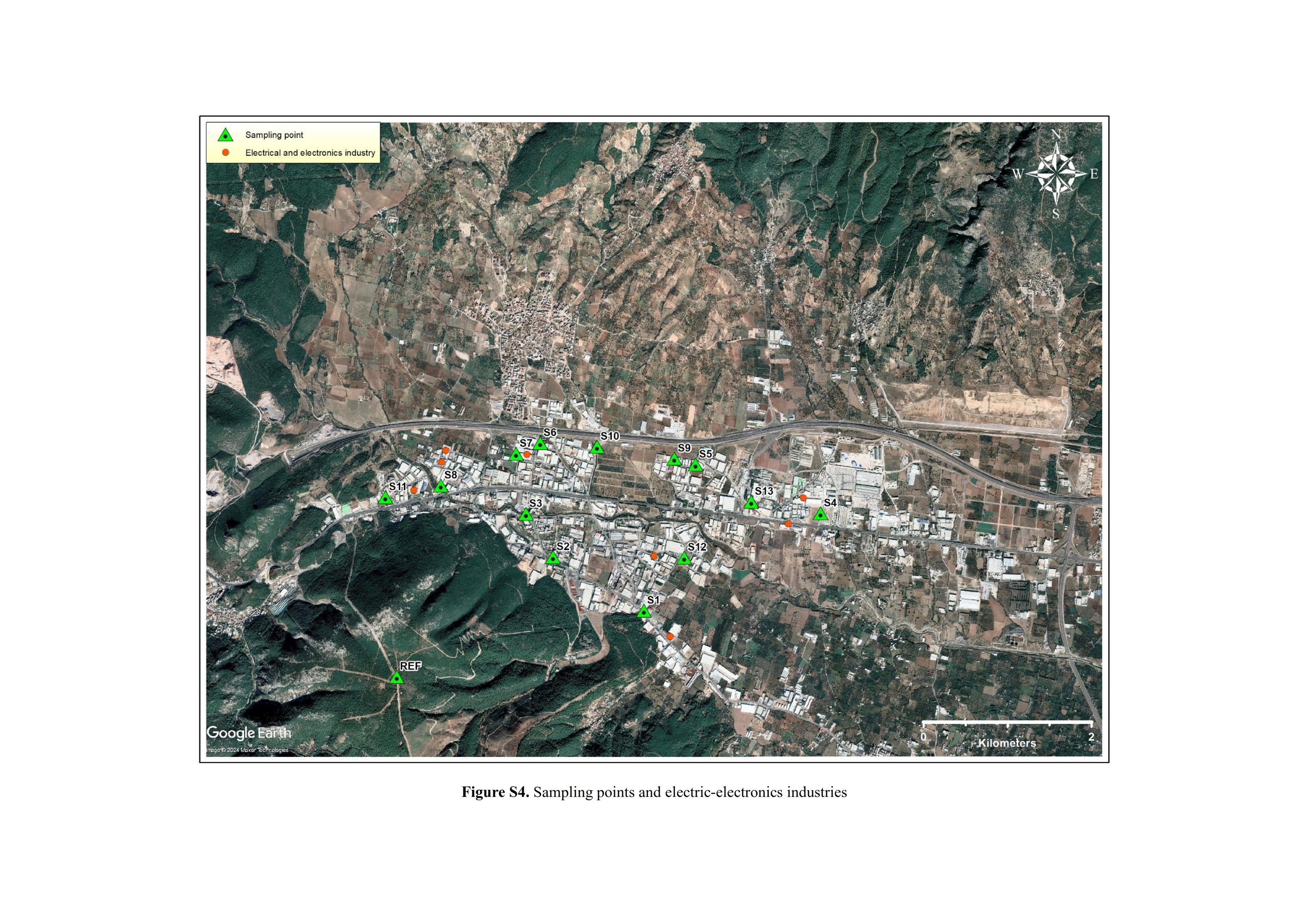

Supplement: Supplemental Information 43 [file peerj-13-20374-s043.jpg]

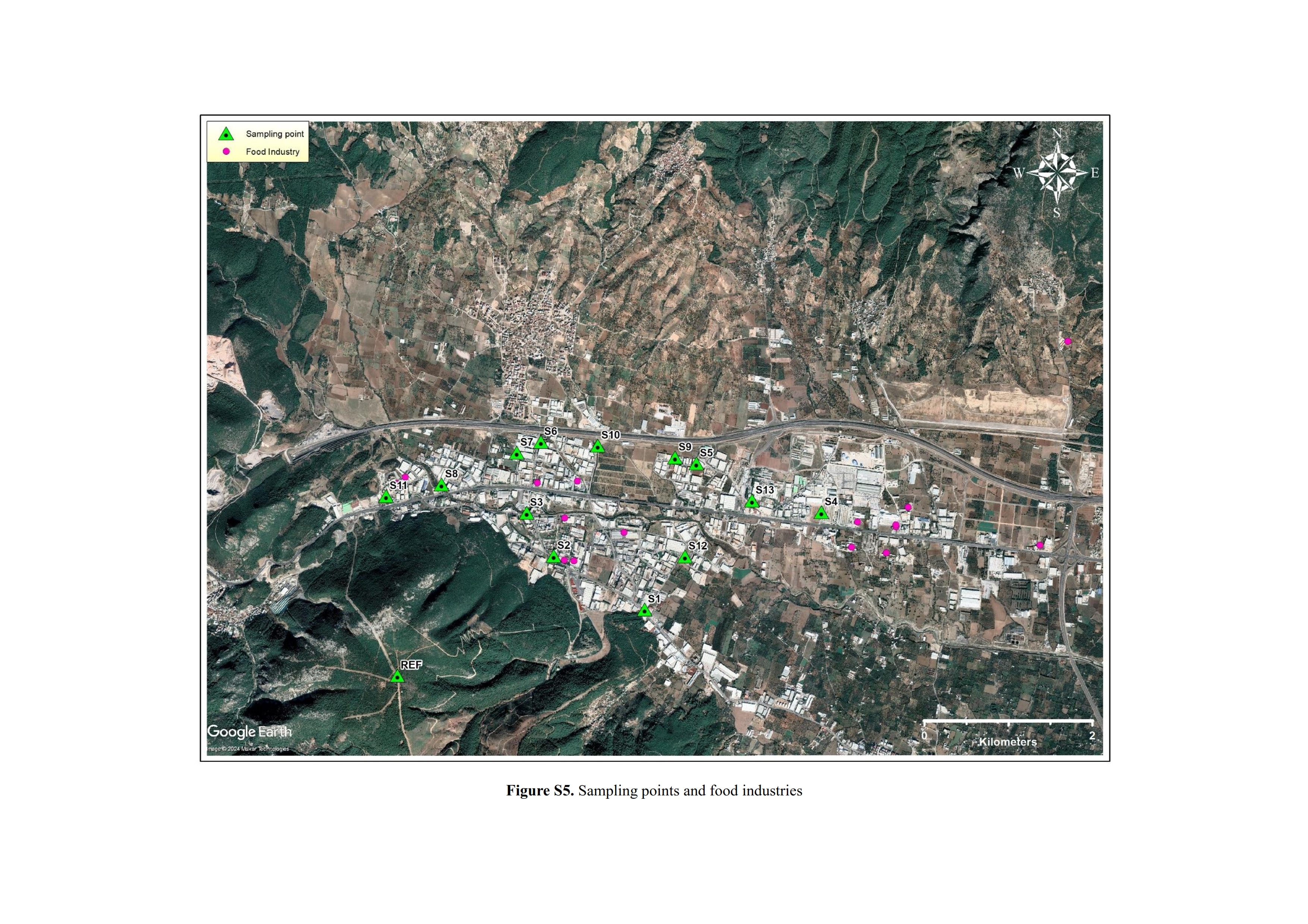

Supplement: Supplemental Information 44 [file peerj-13-20374-s044.jpg]

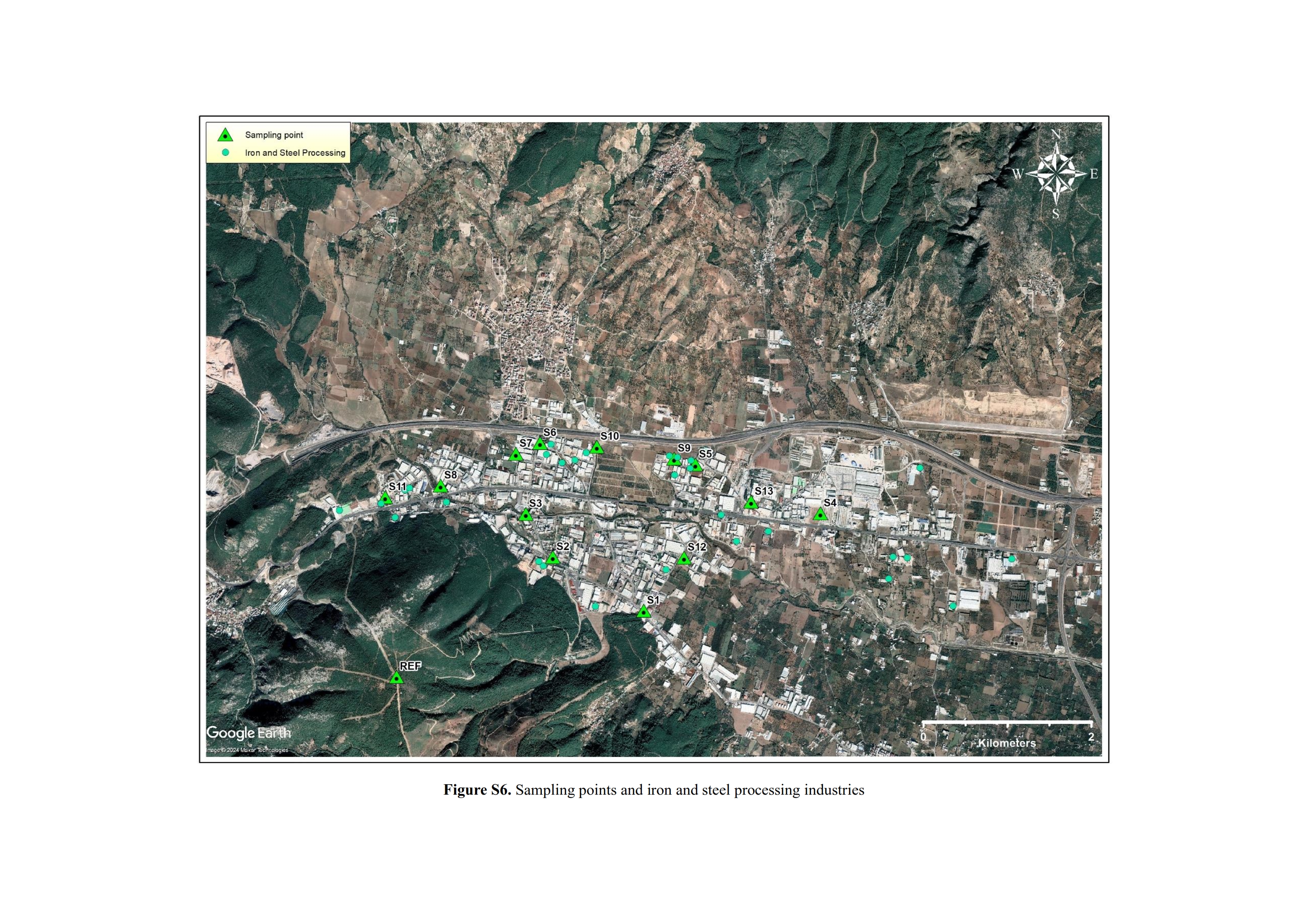

Supplement: Supplemental Information 45 [file peerj-13-20374-s045.jpg]

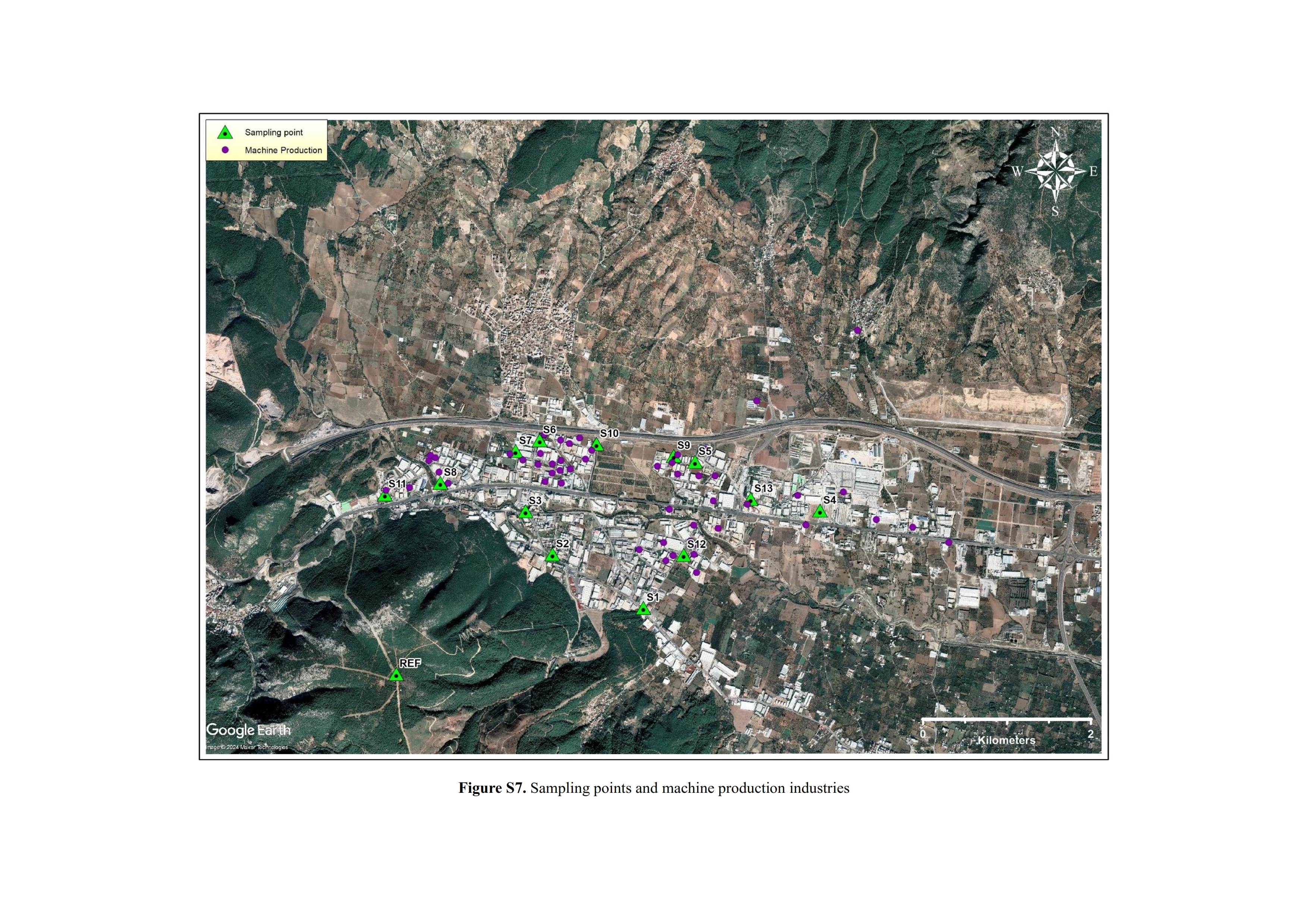

Supplement: Supplemental Information 46 [file peerj-13-20374-s046.jpg]

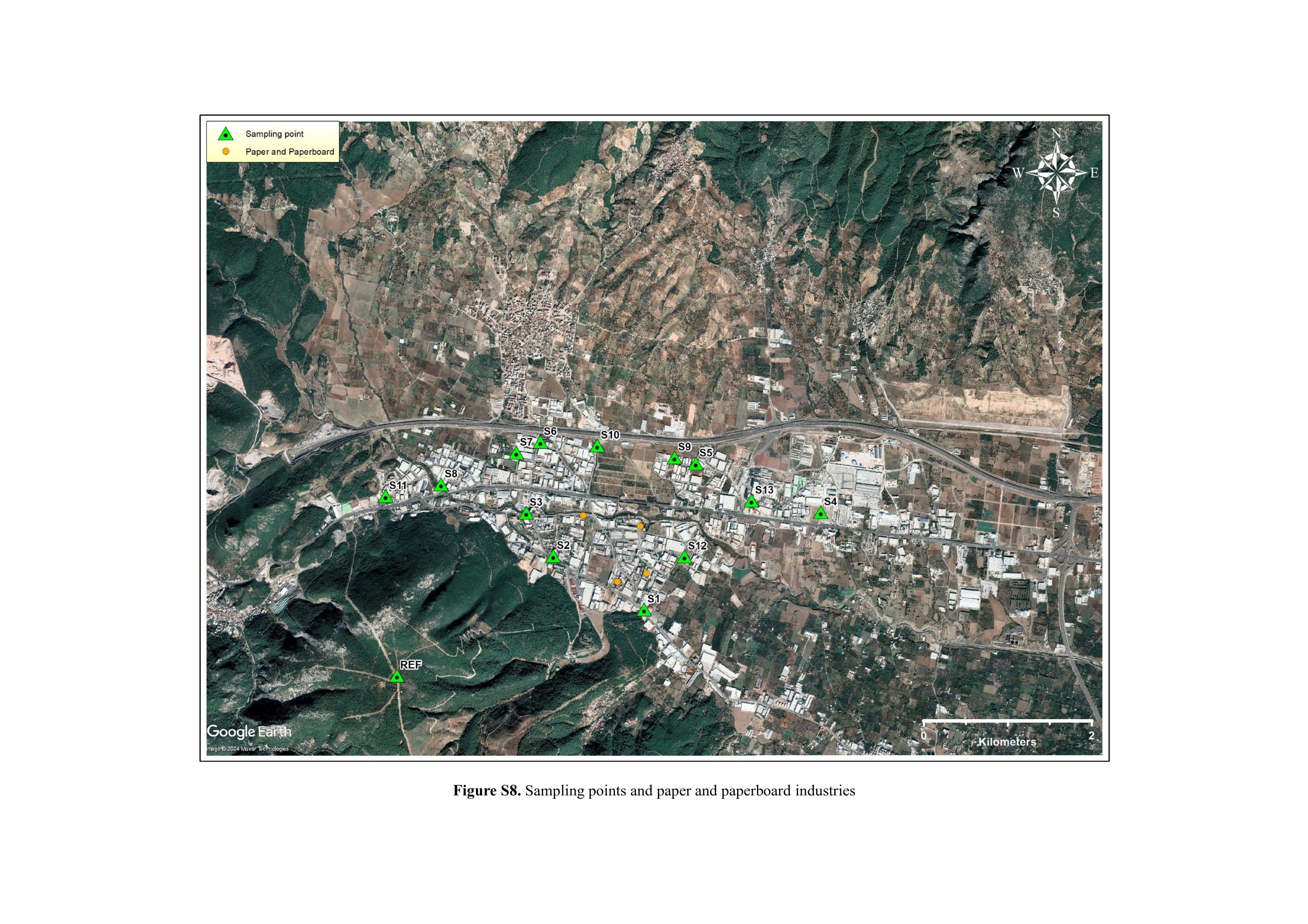

Supplement: Supplemental Information 47 [file peerj-13-20374-s047.jpg]

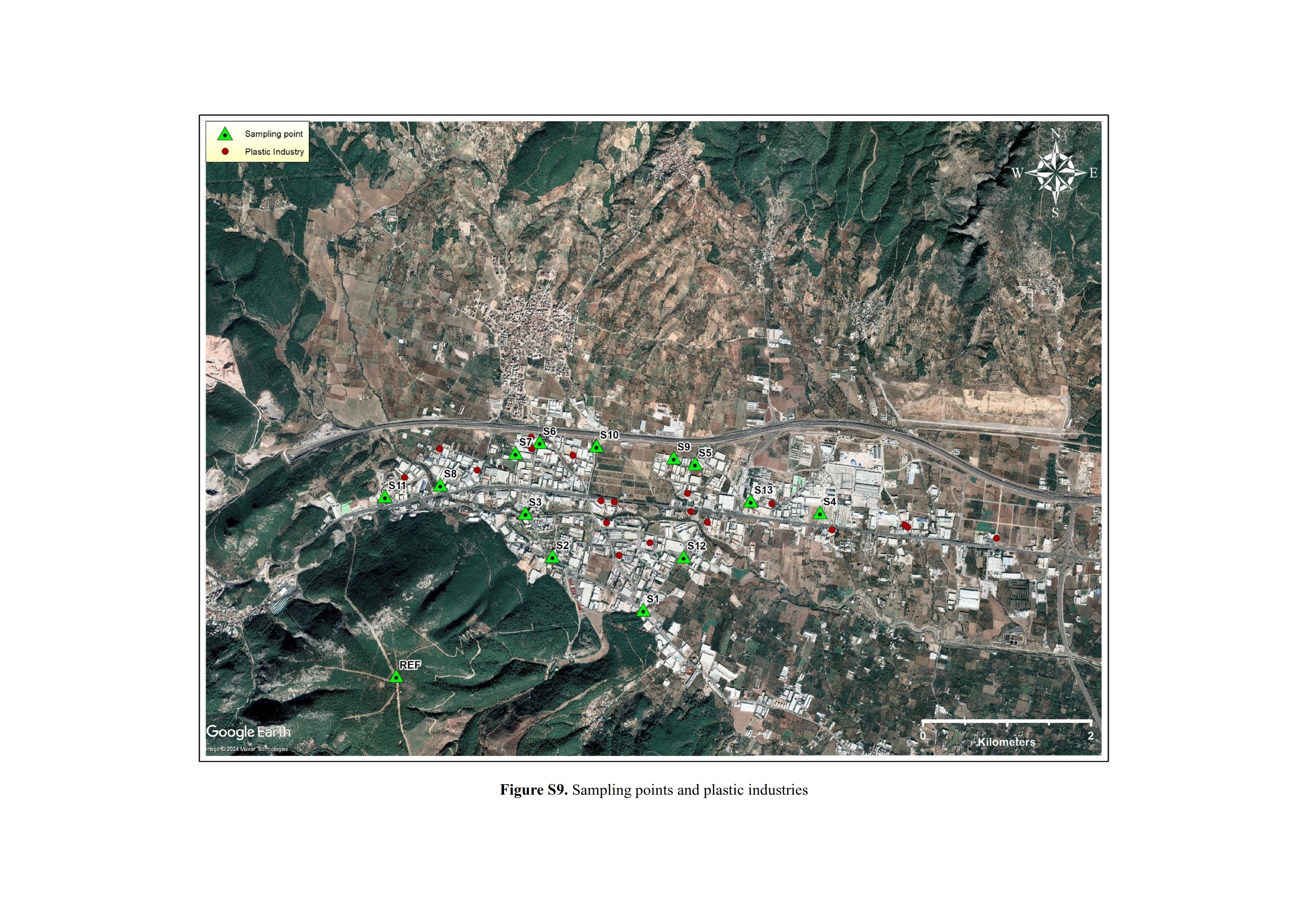

Supplement: Supplemental Information 48 [file peerj-13-20374-s048.jpg]
