# Supplementary material for: Elemental pollution and risk assessment of soils and Gundelia tournefortii in a multi-sector industrial zone with a history of agricultural use
Source: PeerJ. 2025 Nov 24;13:e20374. doi: 10.7717/peerj.20374 (PMC12659707; doi:10.7717/peerj.20374)
Supplement: Supplemental Information 4 [file peerj-13-20374-s004.pdf]

Table S4. Heavy metal levels recorded in soil and *GT* in previous studies (mg/kg$_{dw}$[a])

Site	Media	Cu	Ni	Pb	Zn	Cd	Fe	Cr	Co	Mn	Reference
**Desert, Israel**	**Soil**	3-11.6	10.8-12	<5	0.25-0.44	<1					(Sathiyamoorthy et al., 1997)
	**Plant**	14	3.8	15	30	13	3020			72.7	
	**BCF**[b]	1.21-4.67	0.32-0.35	3	68.18-120	13					
**Cultivated Fields & Roadsides, Türkiye**	**Plant**	0.86			6.6		27.9			8.4	(Turan, Kordali, Zengin, Dursun, & Sezen, 2003)
**Mining Area (Pb), Iran**	**Natural Soil**	10.00	78	67	181	0.2	80				(Chehregani et al., 2009)
	**Waste Pool Soil**	123	98.20	14800	2700	54.1	1400				
	**Plant**	24	8.4	652	820	2.30	1952				
	**BCF**	0.2	0.09	0.044	0.3	0.04	1.4				
**Mining Area (Cu), Iran**	**Growth Soil**	30-450	6-34	22-215	8-220						(Ghaderian & Ghotbi Ravandi, 2012)
	**Leaf**	11	0.6	4	13						
	**BCF**	0.02-0.37	0.02-0.1	0.019-0.18	0.06-1.6						
**Mining Area (Pb), Iran**	**Growth Soil**	162	1730	16700	2950	81.10	1730				(Mohsenzadeh & Rad, 2012)
	**Plant**	24.00	8.40	652.00	820.00	2.30	1952				
	**BCF**	0.15	0.005	0.003	0.28	0.03	1.13				
**Mountain, Türkiye**	**Plant**	27.91		0.04	20.05	0.09	408.37	0.29	1.08		(Tunçtürk et al., 2015)
**Mining Area (Pb&Zn), Iran**	**Growth Soil**		27.5	4384.3	1213.3	51.40					(Moameri et al., 2017)
	**Stems**		20.93	9.46	116.0	0.48					
	**Roots**		23.33	58.11	132.05	1.47					
	**BCF_s**[c]		0.76	0.002	0.095	0.009					
	**BCF_r**[d]		0.84	0.013	0.108	0.028					
**Local Market, Iran**	**Plant**	1.5	1.6		14	0.6	12			8	(Jalali & Fakhri, 2021)
**Erbil Local Market, Iraq**	**Plant**		0.532		3.483		100		0.015	3.37	(Sadee, 2022)

a : dw (dry weight)
b : BCF (Bioconcentration factor for plant)
c : BCF_s (Bioconcentration factor for stem)
d : BCF_r (Bioconcentration factor for root)
